# Restoration of CB1 receptor function in hippocampal GABAergic neurons rescues memory deficits in Huntington’s disease models

**DOI:** 10.1186/s40035-025-00500-w

**Published:** 2025-08-25

**Authors:** Nadia Di Franco, Iker Bengoetxea de Tena, Andrea Sanchez-Ruiz, Alba Pereda-Velarde, Ferran Enfedaque, Candela Gónzalez-Arias, Lluis Maria Miquel Rio, Analia Bortolozzi, Rafael Rodriguez-Puertas, Carlos Costas-Insua, Laura Molina-Porcel, Anna Vazquez-Oliver, Andres Ozaita, Manuel Guzmán, Gertrudis Perea, Silvia Ginés

**Affiliations:** 1 Departament de Biomedicina, Facultat de Medicina I Ciències de La Salut, Institut de Neurociències, Universitat de Barcelona, Barcelona, Spain; 2 Institut d’Investigacions Biomèdiques August Pi I Sunyer (IDIBAPS), Barcelona, Spain; 3 Centro de Investigación Biomédica en Red Sobre Enfermedades Neurodegenerativas (CIBERNED), Instituto de Salud Carlos III, Madrid, Spain; 4 Department of Pharmacology, Faculty of Medicine and Nursing, University of the Basque Country (UPV/EHU), Leioa, Spain; 5 Neurodegenerative Diseases, BioCruces Bizkaia Health Research Institute, Barakaldo, Spain; 6 Instituto Cajal, Spanish National Research Council (CSIC), Madrid, Spain; 7 Department of Neurochemistry and Neuropharmacology, Instituto de Investigaciones Biomédicas de Barcelona (IIBB-CSIC), Barcelona, Spain; 8 Centro de Investigación Biomédica en Red de Salud Mental (CIBERSAM), Instituto de Salud Carlos III, Madrid, Spain; 9 Instituto Universitario de Investigación Neuroquímica (IUIN), Universidad Complutense, Madrid, Spain; 10 Instituto Ramón y Cajal de Investigación Sanitaria (IRYCIS), Madrid, Spain; 11 Alzheimer’s Disease and Other Cognitive Disorders Unit, Neurology Service, Hospital Clínic, I Fundació de Recerca Clínic Barcelona-Institut d’Investigacions Biomèdiques August Pi I Sunyer (FRCB-IDIBAPS), University of Barcelona, Barcelona, Spain; 12 Neurological Tissue Bank, Biobanc-Hospital Clínic-FRCB/IDIBAPS, Barcelona, Spain; 13 Movement Disorders Unit, Neurology Department, Hospital de La Santa Creu I Sant Pau, Biomedical Research Institute Sant Pau (IIB-Sant Pau), 08041 Barcelona, Spain; 14 Department of Medicine and Life Sciences, Universitat Pompeu Fabra, Barcelona, Spain; 15 Present Address: Center for Molecular Biology, Universität Heidelberg (ZMBH), Heidelberg, Germany

**Keywords:** Huntington´s disease, R6/1 mice, Memory decline, CB1 receptor, Hippocampus, GABAergic interneurons

## Abstract

**Background:**

Dysregulation of the endocannabinoid system (eCBS) and the loss of CB1 receptors (CB1R) in the basal ganglia are well-established hallmarks of Huntington’s disease (HD). As a result, significant research efforts have focused on targeting the eCBS to alleviate motor disturbances associated with the disease. Beyond its role in motor control, the eCBS is a complex signaling network critically involved in regulating learning and memory. Despite this, the potential involvement of eCBS dysfunction in the cognitive decline characteristic of HD, often manifested well before motor dysfunction, has remained largely unexplored.

**Methods:**

CB1R expression in the hippocampus was evaluated in both human HD samples and HD mouse models (R6/1 and Hdh^Q7/Q111^ models, including both sexes) using Western blotting, immunohistochemistry, and radioligand binding assays. To restore CB1R function, CB1R agonist WIN-55212–2 was systemically administered, or viral vectors encoding CB1R were locally infused into the hippocampus of HD mice. A multidisciplinary approach combining behavioral, biochemical, electrophysiological, and morphological analyses, was employed to investigate the molecular mechanisms underlying the effects of CB1R activation in the context of HD-related cognitive dysfunction.

**Results:**

In both human HD samples and HD mouse models, CB1R protein levels were reduced in the hippocampus, accompanied by structural synaptic alterations and impairment in spatial, recognition and working memory. Moreover, hippocampal depolarization-induced suppression of inhibition was significantly disrupted in R6/1 mice. Administration of WIN-55212–2 successfully restored these synaptic and cognitive deficits. Immunohistochemical analysis revealed that the CB1R decrease was specifically localized to GABAergic interneurons within the hippocampus. Notably, targeted restoration of CB1R expression in these interneurons via viral vector delivery was sufficient to rescue hippocampal-dependent memory deficits in HD mice.

**Conclusion:**

This study suggests that impaired CB1R function in hippocampal GABAergic interneurons contributes to memory dysfunction in HD.

**Supplementary Information:**

The online version contains supplementary material available at 10.1186/s40035-025-00500-w.

## Background

Huntington’s disease (HD) is a rare, autosomal-dominant neurodegenerative disorder characterized by progressive deterioration of motor, cognitive and emotional functions, caused by a CAG repeat expansion in exon 1 of the *Huntingtin* gene [1–3]. One of the earliest pathological hallmarks of HD, observed in both patients and animal models, is the dysregulation of the endocannabinoid system (eCBS), particularly marked by a loss of CB1 receptors (CB1R) in the basal ganglia. In pre-manifest HD patients, positron emission tomography studies have revealed widespread reductions in CB1R availability [4, 5], while various transgenic HD mouse models consistently show early striatal CB1R loss [6–11]. These observations point to hypoactivation of the eCBS in HD and raise the question of whether cannabinoid-based therapies might help ameliorate disease pathology [8, 9, 12, 13]. Although numerous clinical trials have investigated the efficacy of cannabinoids in alleviating HD-related symptoms, results have been inconsistent, with some reporting improvements while others indicating no benefit or even worsening of motor decline [14–16]. Importantly, cognitive impairments frequently precede motor symptoms by several years in HD patients [17–19]. While these deficits are often linked to executive dysfunction [18, 20, 21], other cognitive domains, particularly those mediated by extra-striatal structures such as the hippocampus, are increasingly recognized as critical contributors. Indeed, significant hippocampal atrophy and spatial memory deficits have been documented in HD patients even before the onset of motor symptoms [22–24]. Corresponding evidence of hippocampal pathology and related cognitive impairments has also been observed in several HD mouse models [25–32]. Notably, CB1Rs are abundantly expressed in memory-related brain regions, including the hippocampus [33–35], where they are predominantly localized to GABAergic interneurons [34–36]. CB1R activation, via endogenous or exogenous ligands, plays a key role in regulating learning and memory processes by retrogradely modulating synaptic transmission [37–39]. In this study, we employed a combination of biochemical, behavioural, and electrophysiological approaches to investigate whether CB1R hypofunction contributes to cognitive deficits in HD. Our findings aim to provide novel insights into the molecular basis underlying synaptic and memory dysfunction in HD, providing a foundation for the development of cannabinoid-based therapies to improve cognitive outcomes and quality of life in HD patients.

## Materials and methods

### Postmortem brain tissues

Human post-mortem samples derived from hippocampus of patients with HD grade 2, 3 and 4 were obtained from the Neurological Tissue Bank, Biobanc-Hospital Clínic-IDIBAPS, Barcelona, Spain. Neuropathologic examination was performed according to standardized protocols [40]. All ethical guidelines contained within the latest Declaration of Helsinki were taken into consideration, and informed consent was obtained from all subjects under study (CER022523 from Bioethics Comission of the University of Barcelona and IMP-039 from Ethics Committee for Research with Medicines-Hospital Clinic/IDIBAPS). Genotype, Vonsattel stage, sex and age information are provided in Table 1.

**Table 1:** Characteristics of the human hippocampal samples used for CB1R immunostaining

Patient ID	Age	Sex	Genotype CAGs	Vonsatel grade	AD-NC	PMD (hours:minutes)
Control 1	74	M	< 25	0	A0B1C0	6:00
Control 2	56	F	< 25	0	A1B1C0	14:00
Control 3	73	M	< 25	0	A0B1C0	6:30
HD 2	62	M	42	2	A1B1C0	14:30
HD 3	59	F	41	2	A1B0C1	4:55
HD 4	86	F	40	2	A3B2C2	12:20
HD 6	76	M	41	2	A3B3C2	6:00
HD 4	59	M	47	4	A0B1C0	5:30
HD 5	72	F	42	3	A0B0C0	7:00
HD 6	68	M	44	3	A0B0C0	4:00

The genotype includes the largest allelic CAG repeat in the *HTT* gene for each individual. AD-NC, Alzheimer´s Disease Neuropathological Change. PMD, postmortem delay

### Animals

R6/1 heterozygous transgenic mice, which express a pathogenic fragment of human mutant *Huntingtin* (*mHtt*) exon 1 with CAG repeats ranging from 115 to 145, were obtained from The Jackson Laboratory (Bar Harbor, ME; #006471) [41]. These mice were maintained in a BL/6:CBA genetic background, alongside their non-transgenic wild-type (WT) littermates that served as controls.

Hdh^Q7^ WT mice, with 7 CAG repeats, and Hdh^Q111^ knock-in (KI) mice, which have a targeted insertion of 109 CAG repeats extending the glutamine segment in murine huntingtin to 111 residues, were maintained on a C57BL/6 genetic background [42]. Hdh^Q7/Q111^ heterozygous mice were intercrossed to generate age-matched heterozygous Hdh^Q7/Q111^ and Hdh^Q7/Q7^ WT littermates.

All mice used in the study were males and females, housed together in numerical birth order in groups of mixed genotypes. Data were recorded for analysis by microchip number. All animals were housed in a colony room with ad libitum access to food and water, maintained at 19–22 °C with 40%–60% humidity, and kept on a 12:12 h light/dark cycle.

The R6/1 mice exhibit motor coordination deficits starting at 15–16 weeks of age [43–45], with impairments in recognition and spatial memory observed by 12 weeks [29]. Hdh^Q111/Q7^ heterozygous KI mice show a decline in memory (recognition, associative and spatial memories) starting at 6 months of age, with motor coordination deficits emerging at 8 months [26, 46, 47].

### Protein extraction and Western blot analysis

Proteins were extracted from brain samples and quantified for Western blot as previously described [26, 29]. Briefly, animals were sacrificed by cervical dislocation at various HD disease stages. The brains were quickly removed, dissected, flash-frozen in dry ice, and stored at − 80 °C until further use. Tissue was homogenized by sonication in lysis buffer containing 150 mmol/L NaCl, 20 mmol/L Tris–HCl (pH 8.0), 50 mmol/L NaF, 1% NP-40, 10% glycerol, supplemented with 1 mmol/L sodium orthovanadate and a protease inhibitor mixture (Sigma-Aldrich). The homogenates were centrifuged at 16,000 × *g* for 15 min at 4 °C, and the supernatant was collected. Protein concentration was determined using the Detergent-Compatible Protein Assay kit (Bio-Rad, Hercules, CA) according to the manufacturer’s instructions.

For Western blotting, protein extracts (15–20 μg) were denatured in SDS sample buffer (62.5 mmol/L Tris–HCl pH = 6.8, 2% (*w*/*v*) SDS, 10% glycerol, 140 mmol/L ß-mercaptoethanol, and 0.1% (*w*/*v*) bromophenol blue) and boiled at 100 °C for 5 min. The denatured samples were resolved on 8%–15% polyacrylamide gels (SDS-PAGE) at 30 mA for 1 h. Proteins were transferred to a nitrocellulose membrane (Amersham® by GE Healthcare Life Sciences®) for 1.5 h at 100 V at 4 °C to prevent excessive warming. Membranes were then incubated for 1 h in blocking buffer (10% non-fat powdered milk in TBS-T: 50 mmol/L Tris–HCl, 150 mmol/L NaCl, pH 7.4, 0.05% Tween 20). Following this, membranes were incubated overnight at 4 °C with primary antibodies: (1:1000; Cat. #CB1-GP-Af530-1, Frontier Institute, Ishikari, Japan), anti-CB2R (1:1000; Cat. #101550 Cayman Chemical, Ann Arbor, MI), anti-PSD95 (1:2000, Cat. #34505, Cell Signalling, Danvers, MA), anti-BDNF (1:500, Cat. #OSB00035W, Osenses, Keswick, Australia), anti-EM48 (1:500, MAB5374, Merck Millipore, Burlington, MA), anti-β-actin (1:1000, Cat. #Clon 4, MP Biomedicals, Irvine, CA). Membranes were then rinsed three times for 10 min each with TBS-T and incubated for 1 h at room temperature with the appropriate horseradish peroxidase-conjugated mouse (Cat. #W402B) or rabbit (Cat. #W401B) secondary antibody (1:3000; Promega, Madison, WI). After washing for 30 min with TBS-T, membranes were developed using the enhanced chemiluminescence (ECL) kit (Cat. #sc-2048, Santa Cruz Biotechnology, Dallas, TX) and imaged with a ChemiDoc imaging system (Bio-Rad). Band intensity was quantified using ImageLab® Software Version 6.0 (2017), and normalized to actin intensity on the same membranes, ensuring that measurements fell within the linear detection range of the ECL reagent. Data are expressed as the mean ± SEM of band density.

### Real-time quantitative PCR (RT-qPCR)

Total RNA was extracted from hippocampal samples of 8-, 12-, and 20-week-old WT and R6/1 male mice using the RNeasy Lipid Tissue Mini Kit (Cat. #74804, QIAGEN, Hilden, Germany). RNA concentration and quality were evaluated with the NanoDrop 1000 spectrophotometer (Thermo Fisher). A total of 500 ng of purified RNA per µL was reverse transcribed using the High-Capacity cDNA Reverse Transcription Kit (Cat. #4374966, Applied Biosystems, Waltham, MA). cDNA synthesis was carried out in a T100 Thermal Cycler (BioRad) at 25 °C for 10 min, 37 °C for 120 min, and 85 °C for 5 min, in a final volume of 20 µL. The resulting cDNA was analyzed by quantitative RT-qPCR in a final volume of 12 µL on 96-well plates (Applied Biosystems) using Premix Ex Taq (Cat. #RR390A, Takara Biotechnology, Kusatsu-shi, Shiga, Japan). Quantitative RT-qPCR was conducted using the StepOnePlus Real-Time PCR system (version 3.0.1) with the following thermal cycling conditions: an initial denaturation at 95 °C for 30 s, followed by 40 cycles of 95 °C for 5 s and 65 °C for 20 s. All samples were analyzed in duplicate. Commercially validated primers were obtained from Integrated DNA Technologies (IDT®): *Cnr1*(Mm.PT.58.30057922) and *Actb* (Mm.PT.39a.22214843.g), the latter serving as housekeeping control. Relative changes in gene expression were calculated using the 2^–ΔΔCT^ method.

### Brain processing and immunohistochemistry

#### Mouse brain tissue preparation

WT and R6/1 mice were deeply anesthetized with ketamine and xylazine (i.p injection; 100 mg/kg and 10 mg/kg, respectively) and immediately perfused transcardially with saline, followed by 4% paraformaldehyde in phosphate buffer. Brains were then removed, post-fixed overnight in the same solution, cryoprotected by immersion in 30% sucrose, and frozen in dry ice-cooled methylbutane. Serial coronal cryostat sections (30 μm) were collected through the entire brain in PBS as free-floating sections and processed as described previously [47].

#### Immunohistochemistry

Sections were rinsed three times in PBS, permeabilized, and blocked in PBS containing 0.3% Triton X-100 and 3% normal goat serum (Pierce Biotechnology, Rockford, IL) for 15 min at room temperature. After washing in PBS, sections were incubated overnight at 4 °C with primary antibodies: CB1R (1:1000; Cat. #CB1-GP-Af530-1, Frontier Institute, Ishikari, Japan), CB2R (1:1000; Cat. #101550 Cayman Chemical, Ann Arbor, MI), GAD67-gt (1:1000, Cat. #MA5-27817, Invitrogen, Waltham, MA), and anti-calbindin D-28 k (1:100, Cat. #CB300, Swant, Burgdorf, Switzerland). Secondary antibodies (Alexa Fluor 555, Cat. #115–565-144; Alexa Fluor 488, Cat. #115-545-152; and Alexa Fluor 647, Cat. #115–605-147; 1:200, Jackson Immunoresearch, West Grove, PA) were applied the following day. Nuclei were counterstained with 4',6-diamidino-2-phenylindole (DAPI, 1:5000, D9542-10MG, Merck) for 10 min. As negative controls, some sections were processed without primary antibody, and no signal was detected in these controls.

#### Human brain tissue preparation

Anterior and middle-posterior hippocampal regions from half human brain tissue samples of control and HD patients (grade 2, 3 and 4) were embedded in paraffin and sectioned at 4 µm. The sections were processed using a BOND-MAX Automated Immunohistochemistry Stainer (Leica Biosystems Melbourne Pty Ltd, Melbourne, Australia). Tissues were deparaffinized and pre-treated with the Epitope Retrieval Solution 2 at 98 °C for 40 min. After this, sections were incubated for 60 min with anti-CB1R antibody (1:1000, Cat. #IMG-3C2**,** ImmunoGenes, Budakeszi, Hungary). Following primary antibody incubation, tissue sections were incubated with the polymer detection system (Cat. #36000, Thermo Scientific Pierce IHC Detection Kit, Waltham, MA) for 8 min, then developed with chromogenic 3,3′-diaminobenzidine using Thermo Scientific Pierce DAB Substrate Kit (Cat. #PI34002) for 10 min, producing a brown precipitate at antigen sites. Finally, hippocampal sections were counterstained with cresyl violet.

### Detection and quantification of EM48 mutant huntingtin (mHtt) aggregates

Hippocampal coronal sections from vehicle- and WIN-treated R6/1 mice were first incubated for 30 min in ammonium chloride (NH_4_Cl) to quench endogenous fluorescence. They were then blocked for 1 h in PBS containing 0.3% Triton X-100 (Merck), 3% normal goat serum (Abcam), and 1% bovine serum albumin (BSA; Merck). After blocking, the sections were incubated overnight at 4 °C with anti-EM48 (1:1000, Merck). The EM48 antibody specifically recognizes huntingtin-containing nuclear aggregates by targeting the first 256 amino acids of human huntingtin protein [48]. Following incubation, sections were washed three times with PBS and then incubated for 2 h at room temperature with Alexa Fluor 488-conjugated AffiniPure donkey anti-mouse IgG (H + L) (1:500, Cat. #715–545-150, Jackson Immunoresearch). Subsequently, the sections were incubated for 10 min with DAPI, mounted with Mowiol, and prepared for imaging. Three coronal hippocampal sections spaced 240 μm apart, were selected from each animal for analysis.

Imaging was performed using a Leica Confocal SP5 with a 63 × oil-immersion objective, ensuring consistent imaging conditions (pinhole size:1 AU, resolution: 1024 × 1024 pixels, and intensity). Automated quantification of nuclear huntingtin aggregates, including the number, intensity, and pixel area of EM48-stained nuclei in the hippocampus, was carried out using the ImageJ particle analysis plugin.

The density of aggregates was calculated as the ratio of the number of positive particles (NII) to the pixel area (mm^2^). EM48 staining index (SI) was determined as the product of the mean number of stained nuclei and the average nuclear stain intensity, as described previously [49].

### Confocal microscopy and quantification

#### Mouse brain tissue

Stained coronal sections were examined using a Carl Zeiss LSM880 confocal microscope, a 10 × , 40 × and 63 × oil-immersion objective, 4 × digital zoom, and a standard Airy disc pinhole. For each mouse, at least three 30-µm thick coronal sections containing the hippocampal region were analyzed. Up to three representative fields were obtained from each slice. In each field, the entire three-dimensional stack of images was obtained using the Z drive present in the Leica microscope, and the size of the optical image was 0.5 μm, with a separation of 2 μm between each one. The CB1R-, calbindin- and GAD67-integrated density was quantified with NIH ImageJ2 version 2.9.0/1.53t (Wayne Rasband, NIH). For double-positive quantification, Pearson’s correlation coefficient was calculated using ImageJ2. A correlation coefficient of 0 indicates no correlation, − 1 indicates perfect anti-correlation, and + 1 indicates perfect correlation.

#### Human brain tissue

For CB1R quantification, hippocampal subregions were defined using the QuPath software [50], following the guidelines from the Allen Brain Atlas. CB1R signal intensity was analyzed with NIH ImageJ2 version 2.9.0/1.53t (Wayne Rasband, NIH). A region of interest (ROI) was manually drawn to cover the extent of the dentate gyrus (DG), CA4, CA3, CA2 and CA1 regions. Integrated density values for the total hippocampus, as well as for the CA1, CA3 and DG subregions, were analyzed for each image, with genotype blinded to the analysis. For each individual, three sections were analyzed, and the mean value from all sections was presented. Histograms represent the mean integrated density for each region.

### Pharmacological treatments

WIN 55212–2 (WIN) (Sigma-Aldrich, Cat. #CAS131543-23-2) was dissolved in dimethylsulfoxide (DMSO, Sigma Aldrich, Cat. #D2650) to a stock concentration of 100 mmol/L and stored at − 20 °C. Appropriate dilutions were made in saline (0.9% NaCl) and Tween 80 (Sigma-Aldrich, Cat. #P8192) before use. For intraperitoneal (i.p.) injections, the DMSO content injected was kept below 0.6 µL per mouse. Control animals received equivalent volumes of vehicle (10 mL/kg).

Animals were treated with daily i.p. injections of WIN (1 mg/kg) for 10 consecutive days (subchronic treatment) before the start of testing and until sacrifice. This protocol was based on previous studies showing that chronic treatment with WIN in adult rats did not affect performance in novel object recognition test or open field test [51].

### Viral vectors and intra-hippocampal stereotaxic delivery

To selectively induce CB1R expression in GABAergic neurons, we generated a plasmid for AAV8-mDlx5/6-CNR1. Infectious AAV viral particles were developed by the Viral Vector Production Unit (UPV) of the Universitat Autònoma de Barcelona (UAB). WT and R6/1 mice, aged 10 weeks, were anesthetized with ketamine hydrochloride/xylazine hydrochloride (i.p.; 100 and 10 mg/kg, respectively, Merck) and injected bilaterally in the hippocampus with AAV8-mDlx-CB1R or AAV8-mCherry (Vector Biolabs) (viral concentrations ranging from 1.8 × 10^9^ to 1.5 × 10^9^). The injection coordinates (in mm) from bregma (anteroposterior and lateral) and from skull (dorsoventral) were as follows: anteroposterior: − 2.0; lateral ± 1.25, and dorsoventral: − 1.3 (CA1). AAVs were injected for over 2 min, and the cannula was left in place for an additional 5 min to allow complete diffusion of the virus before being slowly retracted. Mice were monitored for 1 h post-injection and then returned to the housing facility for 3 weeks. To verify correct CB1R expression, mice were transcardially perfused with paraformaldehyde. The brain tissue was processed and stained as described in the Brain Processing and Immunohistochemistry section. For double-positive quantification, Pearson’s correlation coefficient was calculated using ImageJ2. Behavioral experiments were performed 3 weeks after injection.

### Behavioral assessment

R6/1 and WT mice, as well as mutant Hdh^Q7/Q111^ KI and WT Hdh^Q7/Q7^ mice, underwent a behavioral test battery to assess several neurological phenotypes, as previously described. All behavioral evaluations were performed by experienced investigators who were blinded to the treatment and/or genotype of the animals.

#### Y-maze

Spontaneous working memory was assessed using the Y-maze test. Mice were habituated to the testing room 1 h prior to the session. During the 8-min trial, each mouse was placed in the center of the maze and allowed to move freely. An arm entry was recorded when all four paws of the mouse were within the arm. The apparatus was cleaned thoroughly after each trial. Alternation was defined as successive entries into all three arms in an overlapping triplet sequence. Spontaneous alternation was calculated as the ratio of actual alternations to the total possible alternations, and the result was expressed as a percentage.

#### Novel object location task (NOLT)

NOLT was conducted in a quadrangular open field (40 cm × 40 cm, 40 cm high) with a light intensity of 40 lx at controlled room conditions (19–22 °C, 40%–60% humidity). Mice were first habituated to the arena, which was devoid of objects but included spatial cues attached to the walls, for 15 min per session over two consecutive days. During this phase, spontaneous locomotor activity, as well as the time and the distance spent in the periphery versus the center, were measured. On the third day (NOLT training), two identical objects (A and A′) were placed in adjacent corners of the arena, and mice were allowed to explore for 10 min. Twenty-four hours later (NOLT testing), the object A was placed in the same location, while a new object (A″) was placed in the diagonally opposite corner. During the testing phase, mice were allowed to explore for 5 min. The arena was thoroughly cleaned between trials to eliminate residual odors. Animal movements were tracked and recorded using the SMART® Junior software (Panlab, Spain). Exploration times were recorded to calculate the discrimination index as follows:

$${\text{discrimination}}\;{\text{ index}}\;{ } = \;\frac{{{\text{Time}}\;{\text{ exploring}}\;{\text{ novel}}\;{\text{ object}} - {\text{Time }}\;{\text{exploring }}\;{\text{familiar }}\;{\text{object}}}}{{{\text{Time }}\;{\text{exploring }}\;{\text{both }}\;{\text{objects}}}} \; \times \;100{\text{\% }}$$

#### Novel object recognition task (NORT)

Exploration in the NORT followed the same conditions as described for the NOLT. Mice were first habituated to the arena, without objects, for 15 min on two consecutive days. During this phase, spontaneous locomotor activity and time/distance spent in the periphery versus the center of the arena were measured. On the third day (NORT training), mice were allowed to explore two identical objects (B and B′) for 10 min before being returned to their home cage. Twenty-four hours later (NORT testing), one of the familiar objects (B) and a new object (C) were placed in the same location as during the training session, and the mice were allowed to explore for 5 min. The arena was thoroughly cleaned between trials to prevent odor contamination. Animal movements were tracked and recorded with the SMART® Junior software (Panlab, Spain). Exploration times were recorded and used to calculate the discrimination index as indicated above.

### Golgi-Cox impregnation for dendritic spine analysis

Golgi-Cox impregnation was performed using the Rapid Golgistain Kit (FD Neurotechnologies, Columbia, MD) following the manufacturer's instructions. Mice were euthanized by cervical dislocation, and brains were rapidly removed. The two hemispheres were separated and incubated in a solution of A/B for 2 weeks. After this period, the brains were transferred to solution C for an additional 7 days. The brains were then processed using a cryostat to obtain 100-μm sections, which were mounted on gelatin-coated slides and left to dry for 24 h.

The sections were stained with the solutions provided in the kit and subsequently dehydrated before being mounted with DPX. Bright field images were acquired using a Leica AF6000 epifluorescence microscope with a × 63 numerical aperture objective for dendritic spine analysis. Segments of apical dendrites from hippocampal CA1 pyramidal neurons were selected for analysis. Dendritic spine density was manually quantified using the ImageJ Plug-in Cell Counter. Spines were marked in the appropriate focal plane preventing double counting, and only fully impregnated pyramidal neurons with their soma entirely within the thickness of the section were included. Dendritic segments ranging from 20 to 60 μm in length were analyzed. At least 60 dendrites per group from at least three mice per genotype were counted.

### Radioligand binding assays

Hippocampal membrane homogenates from WT vehicle (*n* = 14), WT WIN (*n* = 10), R6/1 vehicle (*n* = 10) and R6/1 WIN (*n* = 9) mice were prepared for radioligand binding assays. Two independent binding experiments were conducted for each group, with each ligand concentration (0.1 mg/ml) tested in duplicate per experiment, resulting in four technical replicates per data point.

The homogenates were prepared by homogenizing the hippocampal samples with a Teflon-glass grinder in homogenization buffer (1 mmol/L EGTA, 3 mmol/L MgCl_2_, and 50 mmol/L Tris– HCl, pH 7.4) supplemented with 0.25 mmol/L sucrose, at 4 ºC. Synaptosomes were obtained by centrifugation as previously described [52].

For radioligand binding assays, [^3^H]SR141716A (2 nmol/L, Perkin Elmer, Waltham, MA) was used as the ligand. Aliquots of the prepared homogenates were resuspended in a reaction buffer containing 50 mmol/L Tris–HCl, 3 mmol/L MgCl₂, 1 mmol/L EDTA, and 1% BSA (pH 7.4). CP55940 (Sigma-Aldrich) was used as the competitor ligand. The binding reactions were carried out by incubating the tubes containing 0.1 mg/mL protein concentration of mouse hippocampal homogenates for 2 h at 37 °C with agitation. Nonspecific binding was determined by including 10^–4^ mol/L SR141716A in the incubation mixture. After incubation, the reaction was terminated by adding ice-cold wash buffer. The membrane-bound radioligand was collected via vacuum filtration onto Whatman GF/C glass microfiber filter (Sigma-Aldrich®), and radioactivity was quantified using a Packard Tri-Carb 2200CA liquid scintillation counter (PerkinElmer®). Bmax (maximum binding capacity) was determined from saturation binding curves by nonlinear regression, reflecting total receptor binding site density. Statistical analysis was performed using nonlinear regression with the GraphPad Prism software.

### Ex vivo electrophysiological recordings

#### Anesthesia, perfusion and slice preparation

R6/1 and WT mice were anesthetized with pentobarbital and perfused transcardially with ice-cold NMDG-HEPEs recovery solution containing the following (in mmol/L): *N*-methyl-*D*-glucamine 93, KCl 2.5, NaH_2_PO_4_ 1.2, NaHCO_3_ 30, HEPES 20, glucose 25, sodium ascorbate 5, thiourea 2, sodium pyruvate 3, MgSO_4_ 10 and CaCl_2_ 0.5 (pH = 7.3). The brain was then rapidly removed, and coronal hippocampal slices (350-μm thick) were prepared using a Leica Vibratome VT1200S. The slices were incubated at room temperature in artificial cerebrospinal fluid (aCSF) containing (in mmol/L): NaCl 124, KCl 2.69, KH_2_PO_4_ 1.25, MgSO_4_ 2, NaHCO_3_ 26, CaCl_2_ 2, glucose 10 and L( +)-ascorbic acid 0.4, continuously gassed with carbogen (95% O_2_/5% CO_2_, pH = 7.3).

#### Electrophysiological recording setup

For whole-cell patch-clamp recordings of CA1 neurons, slices were visualized using a Nikon Eclipse FN1 microscope equipped with a 40 × water-immersion lens and infrared-DIC optics. Borosilicate glass capillaries (resistance 3–6 MΩ) were filled with an intracellular solution, containing: either (in mmol/L) K-gluconate 135, KCl 10, HEPES 10, MgCl_2_ 1, and ATP–Na_2_ 2 (pH = 7.3 adjusted with KOH) for excitatory synaptic recordings (EPSCs); or K–MeSO_4_ 100, KCl 50, HEPES 10, ATP–Na_2_ 2, and GTP-Tris salt 0.4 (pH 7.2–7.3 adjusted with KOH) for inhibitory synaptic recordings (IPSCs). The electrophysiological recordings were obtained with a PC-ONE amplifier (Dagan Corporation, Tucson, AZ) in the voltage-clamp mode, with a holding potential (*V*_h_) of − 70 mV. Series and input resistances were monitored, and recordings with an access resistance change greater than 20% during the experiment were discarded. The signals were filtered at 1 kHz and acquired at 10 kHz sampling rate. Data acquisition, stimulus generation, and analysis were performed using the pCLAMP 11.2 software (Axon Instruments, Union City, CA). All the experiments were performed at room temperature.

#### Synaptic stimulation and depolarization-induced synaptic plasticity

Synaptic stimulation was performed using theta glass capillaries (2–5 μm tip diameter) filled with aCSF, which were placed in the stratum radiatum. Synaptic currents were evoked by delivering single pulses (250-μs duration) continuously at a frequency of 0.33 Hz. To isolate EPSCs, aCSF was supplemented with picrotoxin (50 μmol/L) and CGP55845 (5 μmol/L). For IPSCs, aCSF was supplemented with CNQX (10 μmol/L) and D-AP5 (50 μmol/L). Depolarization-induced suppression of inhibition (DSI) and excitation (DSE) were evoked by applying depolarizing steps from − 70 mV to 0 mV for 5 and 10 s, respectively. For the DSE protocol, the average of two consecutive synaptic responses was used for analysis, while for DSI protocol, three consecutive responses were averaged.

### Statistics

Data were analyzed using the GraphPad Prism software (version 10.3) and are presented as mean ± standard error of the mean (SEM). The distribution of data was analyzed using the D’Agostino–Pearson and Shapiro–Wilk normality tests. For data that followed a normal distribution, parametric tests were applied: Student's *t*-test (95% confidence) for comparisons between two groups or two-way ANOVA followed by Tukey’s post-hoc test for multiple comparisons. For data that deviated significantly from a normal distribution, non-parametric tests were used, including Mann–Whitney U test for two-group comparisons. *P* < 0.05 was considered statistically significant.

## Results

### CB1R protein levels are decreased in the hippocampus of R6/1 mice

**Fig. 1:**
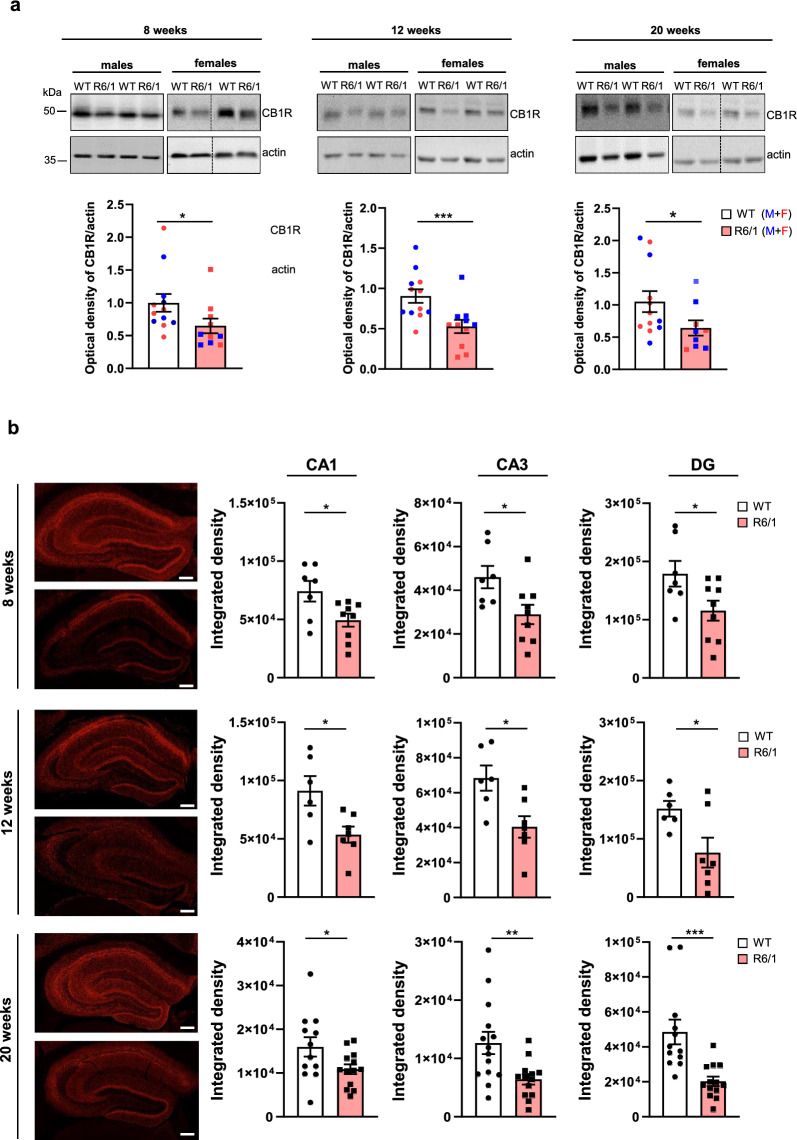
Early reduction of CB1R protein levels in the hippocampus of R6/1 mice. **a** Representative immunoblots and quantitation of CB1R in the total hippocampal lysates from WT and R6/1 mice at different stages of disease progression. Data are presented as mean ± standard error of the mean (SEM) with *n* = 9–12 mice per group. *P < 0.05 ***P < 0.001 vs. WT mice, Mann–Whitney test. **b** Representative confocal microscopic images showing immunofluorescence labeling for CB1R in hippocampal coronal sections from WT (upper) and R6/1 mice (lower) at different stages of disease progression. Mean ± SEM with *n* = 6–12 mice per group. Scale bars, 100 µm. Histograms show the integrated density of CB1R staining. *P < 0.05, **P < 0.01, ***P < 0.001 vs. WT mice, Mann–Whitney test. Male mice were used in this analysis

Early reductions of CB1R expression have been reported in the basal ganglia of both HD patients and mouse models [4, 8, 9, 53, 54]. However, comparatively little is known about CB1R expression in the hippocampus. To address this gap, we evaluated CB1R protein levels in the hippocampus of male and female WT and R6/1 transgenic mice at three stages of disease progression: pre-symptomatic (8 weeks), early symptomatic (12 weeks) and late symptomatic (20 weeks). Our findings reveal a significant reduction of hippocampal CB1R protein levels in R6/1 mice compared to WT controls at all time points analyzed (Fig. 1a). To investigate whether this reduction was driven by transcriptional changes, we performed RT-PCR analysis (Fig. S1). No significant differences in CB1R mRNA expression were observed between genotypes or across disease stages, suggesting that the observed decrease in CB1R protein level is likely mediated by post-transcriptional mechanisms. Finally, to determine whether this CB1R decrease represents a general phenotype associated with mutant huntingtin expression, we also assessed CB1R protein levels in the cerebellum, a brain region that highly expresses CB1R [55] and exhibits significantly less atrophy than the striatum in HD patients [56]. In contrast to the hippocampus, CB1R protein level in the cerebellum remained unchanged (Fig. S2), suggesting that the reduction of CB1R protein level is region-specific rather than global.

Next, we examined whether the observed decrease in total CB1R protein levels was associated with specific hippocampal sub-regions. Hippocampal slices from R6/1 and WT mice were stained for CB1R and analyzed using confocal microscopy. The analysis focused on the stratum pyramidae and stratum radiatum of CA1 and CA3 regions, as well as the granular and polymorphic layers of DG (Fig. 1b). CB1R immunoreactivity was observed in all hippocampal sub-regions, regardless of genotype. However, a significant reduction in CB1R signal was detected in R6/1 mice compared to WT mice starting as early as 8 weeks of age.

### CB1R protein levels are also decreased in the HD human hippocampus

**Fig. 2:**
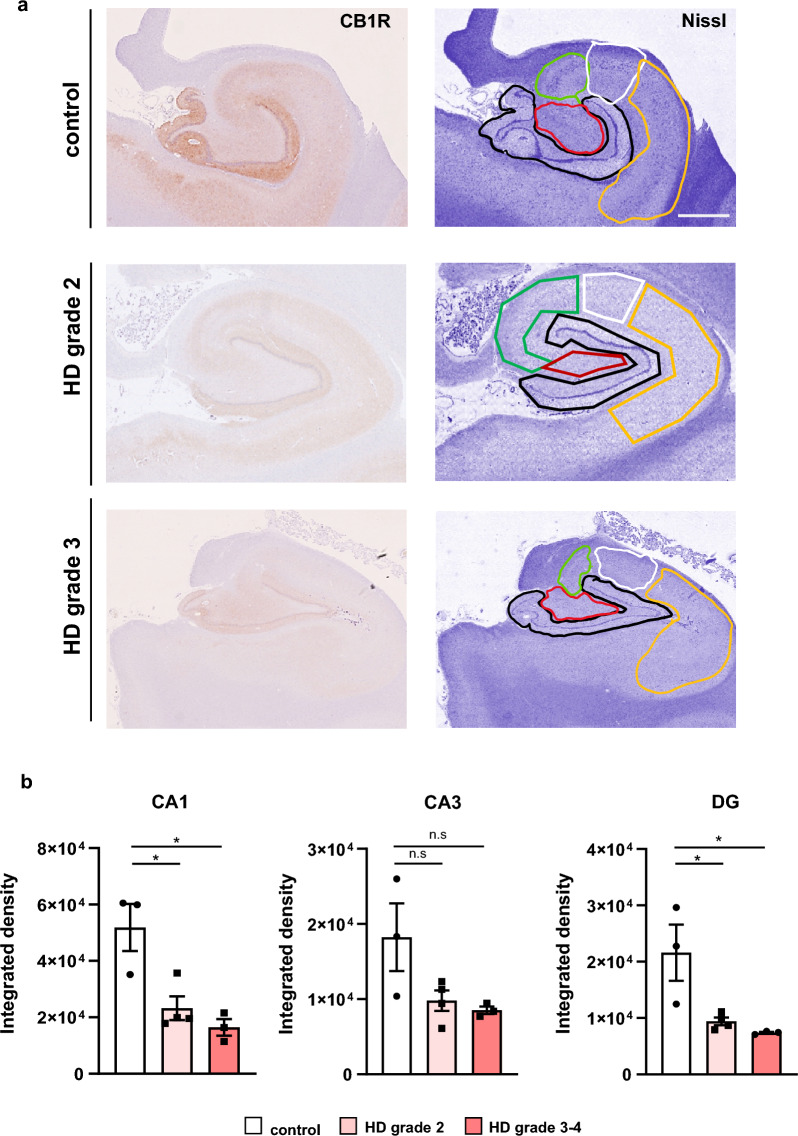
Reduced CB1R protein levels in the hippocampus of human HD patients. **a** Representative immunohistochemical images of CB1R and Nissl staining in postmortem hippocampal sections from control individuals and HD patients at early (Vonsattel grade 2) and late (Vonsattel grade 3–4) disease stages. Nissl staining highlights hippocampal subregions used for CB1R signal quantification: dentate gyrus (black), CA4 (red), CA3 (green), CA2 (white) and CA1 (yellow). **b** Quantification of CB1R intensity (integrated density) within individual hippocampal subregions. Data are shown for control (*n* = 3), early-stage HD (*n* = 4), and late-stage HD (*n* = 3) samples. For each subject, three hippocampal sections were analyzed, and the mean signal intensity was calculated. Data are presented as mean ± SEM. *P < 0.05, **P < 0.01, vs. control, one-way ANOVA followed by Tukey's post-hoc test. Scale bar, 2 mm

To validate the reduction of CB1R observed in HD mouse models, we next examined CB1R expression in postmortem human hippocampal tissues from control individuals and HD patients representing various Vonsattel grades of striatal neuropathology (Fig. 2). CB1R immunoreactivity was evident across all examined hippocampal subregions (Fig. 2a). Compared to controls, HD patients exhibited a significant decrease in CB1R staining throughout the hippocampus, regardless of disease stage. When analyzing individual subregions, a significant reduction in CB1R intensity was detected in both the CA1 and DG areas. Although a downward trend in CB1R level was also observed in the CA3 subregion, this did not reach statistical significance (Fig. 2b). Overall, these findings from both mouse models and human postmortem samples provide evidence for reduced CB1R expression in the HD hippocampus.

### Sub-chronic treatment with WIN improves memory performance of R6/1 mice

Given the observed reduction of CB1R in the R6/1 hippocampus, we hypothesized that pharmacological activation of the remaining CB1Rs could mitigate HD-related memory decline. To test this, we performed a sub-chronic treatment with the CB1R agonist WIN over a 10-day period at two different stages of HD progression: 11 weeks, before cognitive symptoms are apparent and 13 weeks, when cognitive decline has already manifested [29, 30]. It is important to note that the R6/1 mice do not exhibit motor impairments in the fixed rotarod test until 15–16 weeks of age [43, 45]. Initially, we determined the optimal WIN dose in WT mice that did not induce adverse effects on memory performance. Two doses of WIN (1 and 4 mg/kg) were administered intraperitoneally (i.p.) to 13-week-old male and female WT mice, and both memory and motor behavior were evaluated (Fig. S3a). No adverse effects of WIN, at either the low or the high dose, were observed on the total distance traveled during the open field test, or the time spent in the periphery and the center of the open field arena (Fig. S3b). Similarly, no differences were detected in spatial memory, recognition memory, or working memory, as assessed by NOLT, NORT and Y-maze tests, respectively (Fig. S3c–e).

**Fig. 3:**
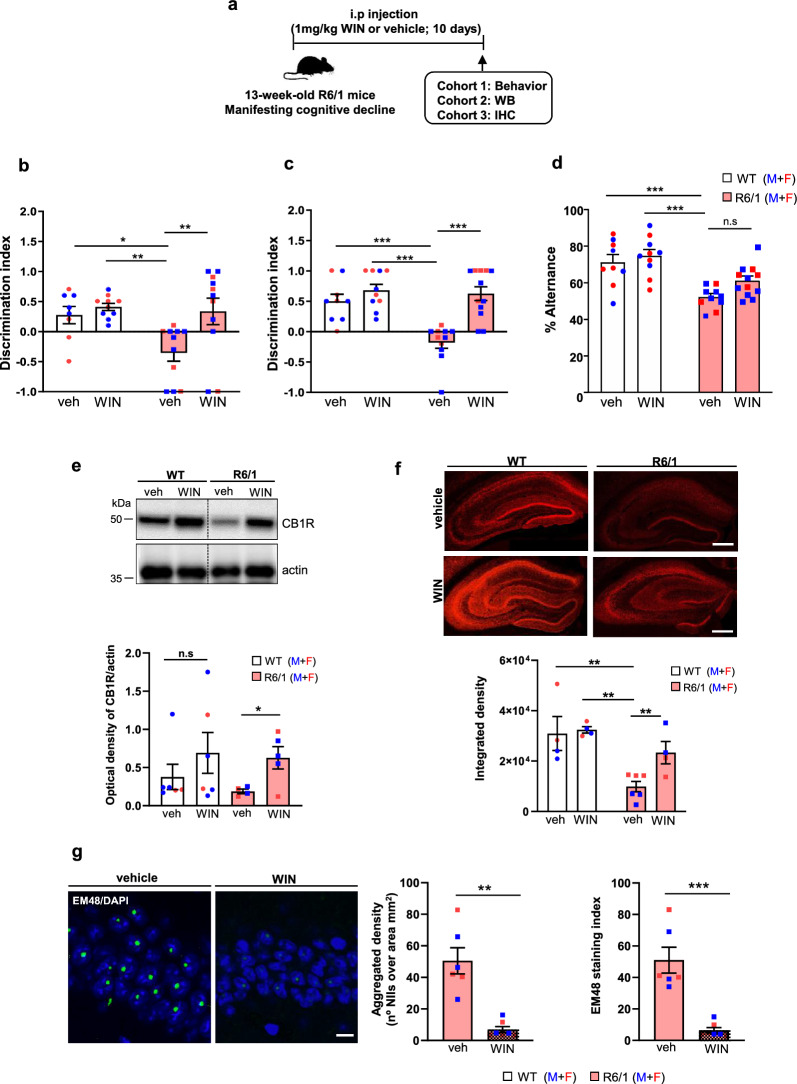
Sub-chronic WIN treatment improves cognitive performance, restores hippocampal CB1R levels and reduces mutant huntingtin aggregates in R6/1 mice. **a** Timeline of the experiments. **b** Performance in the novel object location task assessing spatial memory. Two-way ANOVA revealed significant effects of WIN treatment (*F*(1, 23) = 5.585, *P* = 0.0269) and genotype (*F*(1, 23) = 6.451, *P* = 0.0183). **c** Performance in the novel object recognition task assessing recognitive memory. Two-way ANOVA showed significant effects of WIN treatment (*F*(1, 38) = 21.56, *P* < 0.0001) and genotype (*F*(1, 38) = 12.02, *P* = 0.0013). **d** Performance in the Y-maze task evaluating working spatial memory. Two-way ANOVA showed significant effects of WIN treatment (*F*(1, 37) = 4.498, *P* = 0.04) and genotype (*F*(1, 37) = 29.54, *P* < 0.0001). Data are presented as mean ± SEM. Post-hoc comparisons were conducted using Tukey's test; *P < 0.05, **P < 0.01 and ***P < 0.001. *n* = 8–12 animals per group and genotype. **e** Representative immunoblots and quantification of CB1R protein levels in total hippocampal lysates from vehicle- and WIN-treated WT and R6/1 mice. Data are presented as mean ± SEM, *n* = 4–6 mice per group and genotype. Two-way ANOVA revealed a significant effect of WIN tretment (*F*(1, 17) = 10.17, *P* = 0.0054). Post-hoc comparisons using Tukey's test indicated *P < 0.05. **f** Representative confocal images and quantification of CB1R immunofluorescence in hippocampal coronal sections from vehicle- and WIN-treated WT and R6/1 mice. Scale bars, 100 µm. Mean ± SEM, *n* = 5–7 mice per group and genotype. Two-way ANOVA revealed a significant effect of WIN treatment (*F*(1, 12) = 13.28, *P* = 0.0034) and genotype (*F*(1, 12) = 13.54, *P* = 0.0031). Post-hoc comparisons using Tukey's test indicated **P < 0.01. **g** Representative confocal images of EM48 immunostaining showing nuclear mutant huntingtin inclusions in the hippocampal CA1 region of 14-week-old R6/1 mice. Nuclei were counterstained with DAPI. Scale bar, 25 μm. Histograms show the density of EM48-positive neuronal intranuclear inclusions (NIIs/mm^2^) and the staining index (total number of EM48-positive inclusions per total number of DAPI-stained nuclei). Mean ± SEM, with *n* = 5–7 mice per group. Statistical significance was assessed using the Mann–Whitney test; **P < 0.01, ***P < 0.001

Based on these results, the 1 mg/kg dose was selected for subsequent experiments. First, we evaluated long-term memory using the NOLT and NORT tests. Vehicle-treated R6/1 mice exhibited impaired spatial and recognition memory, as indicated by a reduced discrimination index. Administration of WIN either at the symptomatic cognitive stage (Fig. 3b, c) or at the presymptomatic cognitive stage (Fig. S4b, c), significantly improved location and recognition memory deficits in R6/1 mice, with discrimination indices comparable to those of WT mice. We then evaluated spatial working memory using the Y-maze test (Fig. 3d and Fig. S4d). The vehicle-treated R6/1 mice showed significantly lower spontaneous alternation scores compared to WT mice. Like the long-term memory results, WIN treatment significantly enhanced spontaneous alternation performance in R6/1 mice, demonstrating a beneficial effect on their memory performance.

Next, we validated the positive effects of WIN treatment in Hdh^Q7/Q111^ mutant KI mice, which exhibit a delayed onset and slower progression of HD pathology compared to R6/1 mice [57]. Sub-chronic WIN treatment was administered to male and female WT and KI mice at 6 months of age, when cognitive symptoms, but not motor deficits, are already present [25, 26, 29]. Memory performance was evaluated using the NOLT, NORT and Y-maze tests (Fig. S5). Consistent with the results in R6/1 mice, WIN administration also restored memory deficits in KI mice (Fig. S5b–d). These findings demonstrate that WIN administration, whether at symptomatic or presymptomatic disease stage, exerts beneficial effects on cognitive dysfunction in HD models.

### Hippocampal CB1R levels are restored in HD mice following sub-chronic administration of WIN

To better understand the molecular mechanisms underlying WIN-induced memory improvement in HD mouse models, we first evaluated the levels of CB1R in the hippocampus of vehicle- and WIN-treated WT and R6/1 mice (Fig. 3). Given the similar cognitive benefits observed in R6/1 mice treated at both symptomatic and presymptomatic disease stages, we focused our mechanistic studies on the symptomatic stage (13 weeks of age). CB1R levels were initially measured by Western blot analysis in total hippocampal lysates. Quantitative analysis revealed a reduction in CB1R protein level in vehicle-treated R6/1 mice, which was restored following WIN administration (Fig. 3e). Notably, WIN treatment also significantly modulated CB1R levels in WT mice. These results were corroborated by immunohistochemistry, where WIN-treated R6/1 mice displayed CB1R levels comparable to those of WT controls (Fig. 3f).

Given that WIN treatment also improved memory performance in KI mice, we next investigated whether similar CB1R-related mechanisms contributed to this effect. As a first step, we assessed hippocampal CB1R levels in naive 6-month-old KI mice (Fig. S5e). We found a modest, yet non-significant reduction of CB1R levels in the KI mice compared to WT mice. WIN treatment induced a trend of increase in CB1R levels (Fig. S5f). Finally, to further examine CB1R distribution, we performed immunohistochemistry. Similar to R6/1 mice, a significant reduction in CB1R staining was observed in the hippocampus of vehicle-treated KI mice, which was restored to near WT levels following WIN administration (Fig. S5g). Overall, these findings suggest that the cognitive benefits of WIN may be mediated, at least in part, by normalization of hippocampal CB1R levels in HD mouse models.

### Sub-chronic administration of WIN reduces the aggregation of mutant huntingtin (mHtt) in the hippocampus of R6/1 mice

A key neuropathological hallmark of HD is the aggregation of mHtt in neuronal intranuclear inclusions [1, 58]. To investigate whether WIN could mitigate cognitive decline by reducing mHtt inclusion formation, we performed immunohistochemical analysis using EM48 antibody on coronal hippocampal slices from vehicle- and WIN-treated R6/1 mice (Fig. 3g). As previously described by our group and others [43, 59], EM48-positive intranuclear inclusions were observed in the hippocampus of R6/1 mice. Notably, WIN treatment resulted in a significant reduction in both the density of intranuclear mHtt inclusion and the EM48 staining index. These findings suggest that CB1R activation in R6/1 mice may delay the onset of hippocampal neuropathology and contribute to amelioration of cognitive decline.

### Hippocampal CB1R binding affinity is normalized in R6/1 mice following sub-chronic administration of WIN

Radioligand displacement experiments were conducted using hippocampal membrane homogenates from vehicle- and WIN-treated WT and R6/1 mice. Inhibition curves generated using the radiolabeled, CB1R-selective antagonist [^3^H]SR141716A vs. the non-labeled CB1R agonist CP55940 revealed significantly reduced B_max_ values in vehicle-treated R6/1 mice compared to vehicle-treated WT mice (Fig. S6b and Table 2). Notably, sub-chronic WIN administration restored B_max_ values of R6/1 mice to levels comparable to WT controls, without affecting B_max_ in WT mice (Fig. S6b and Table 2). Total binding of [^3^H]SR141716A, measured as the peak of the inhibition curve, further confirmed reduced CB1R protein level in vehicle-treated R6/1 mice. Notably, WIN treatment significantly increased the total binding in R6/1 mice, consistent with the recovery of CB1R levels (Fig. S6d).

**Table 2:** IC_50_ (nmol/L), B_max_ (fmol/mg prot) and fraction high values of the hippocampal membrane homogenates from WT and R6/1 mice treated with vehicle or WIN (1 mg/kg)

[^3^H]SR141716A (2 nmol/L)			
Group	IC_50_ (nmol/L)	B_max_ (fmol/mg prot)	Fraction high
WT veh	23.24 ± 18.80 1139 ± 19.44	802.0 ± 20.39	0.4696
WT WIN	18.05 ± 16.72 849.60 ± 17.12	828.0 ± 39.64	0.4654
R6/1 veh	64.45 ± 12.12***	668.3 ± 11.56***	0.0659*
R6/1 WIN	10.86 ± 16.03^$$$^ 381.60 ± 31.11^$$$^	830.6 ± 46.32^$$^	0.6378^$$$^

WT veh vs. R6/1 veh (*) and R6/1 veh vs. R6/1 WIN (^$^). Extra-sum-of squares F test, *P < 0.05, ^$$^*P* < 0.01, ***^$$$^*P* < 0.001

Regarding receptor affinity, analysis of the inhibition curves, showing the percentage of [^3^H]SR141716A binding displaced by CP55940, revealed significant differences in IC_50_ values and in the best-fit models (one or two binding sites). The vehicle-treated WT mice displayed a curve indicative of two binding sites (high and low affinity), whereas the vehicle-treated R6/1 mice lacked the low-affinity site and showed a shift toward a single high-affinity binding site. In contrast, WIN-treated R6/1 mice showed a binding profile with both binding sites, similar to WT mice (Fig. S6c and Table 2). These findings suggest that the WIN treatment not only restores CB1R protein level but also reverses conformational alterations in CB1R observed in R6/1 mice.

### Hippocampal CB2R levels remain unaltered in the hippocampus of HD mice

Our results above showed a reduction in CB1R levels in the hippocampus of HD mouse models, which was reversed by WIN treatment. Given that increased CB2R expression has been reported in several pathological conditions, including neurodegenerative diseases such as HD [60–62], we next evaluated hippocampal CB2R levels in naive HD mice. To this end, we analyzed total hippocampal lysates from cognitively symptomatic R6/1 (12 weeks) and KI (6 months) mice using Western blot. No significant differences in CB2R levels were observed compared to WT mice (Fig. S7a, b).

We then examined whether the memory-enhancing effects of WIN treatment were associated with changes in CB2R expression. Unlike the changes seen in CB1R, WIN treatment did not significantly affect CB2R levels in either R6/1 or KI mice (Fig. S7c, d).

### Sub-chronic WIN administration restores structural plasticity and synaptic-related protein expression in R6/1 mice

**Fig. 4:**
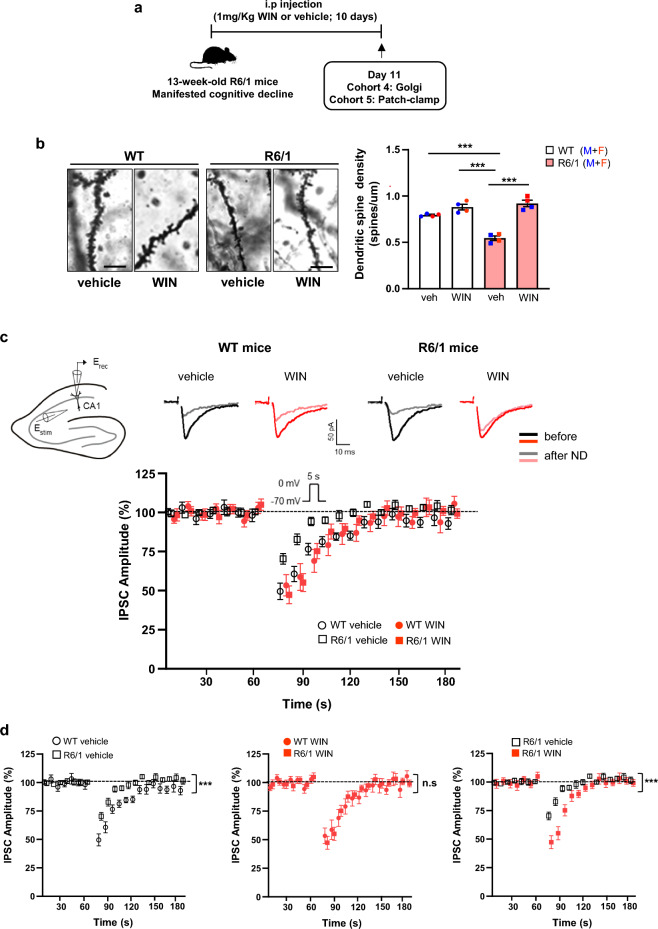
Sub-chronic WIN-55212–2 treatment restores structural plasticity and enhances depolarization-induced suppression of inhibition (DSI) in the hippocampus of R6/1 mice. **a** Timeline of the experiments. **b** Representative photomicrographs of Golgi-stained apical dendrites from CA1 pyramidal neurons in the *stratum radiatum* in vehicle- and WIN-treated WT and R6/1 mice. Right, dendritic spine density (spines/μm) quantified from > 50 dendrites per group (*n* = 4 mice per group and genotype). Two-way ANOVA revealed significant effects of WIN treatment (*F*(1, 12) = 15.72, *P* = 0.0019) and genotype (*F*(1, 12) = 71.53, *P* < 0.0001). Tukey's post-hoc test indicated ***P < 0.001. Scale bar, 5 μm. **c** Representative traces of inhibitory postsynaptic currents (IPSCs) recorded (average from 2 responses) from CA1 pyramidal neurons before and after a 5-s depolarizing step to 0 mV. Lower panel, normalized average IPSC amplitudes before and after depolarization. Vehicle-treated WT (*n* = 13 neurons; 3 mice), vehicle-treated R6/1 (*n* = 18 neurons; 2 mice), WIN-treated WT (*n* = 12 neurons; 5 mice), and WIN-treated R6/1 (*n* = 17 neurons; 4 mice). **d** Normalized average IPSC amplitudes across all groups. Mean ± SEM. Statistical significance was determined using two-way ANOVA with Bonferroni post-hoc test. Both male and female mice were included in all analysis

CB1R signaling plays a critical role in regulating synaptic plasticity and memory by modulating dendritic spine density and expression of synaptic-related proteins [63, 64]. Since cognitive deficits in R6/1 mice are associated with reduced dendritic spines in the hippocampus [26, 65], we next analyzed structural plasticity by measuring apical dendritic spine density in CA1 pyramidal neurons. As expected, R6/1 mice exhibited a significant reduction in spine density compared to WT mice, a reduction that was reversed following WIN treatment (Fig. 4b).

Given that key molecular components of synaptic plasticity and neuronal function are known to be downregulated in the R6/1 hippocampus [43, 63, 64], we next investigated whether WIN-induced memory improvement was associated with restoration of synaptic-related protein expression. Specifically, we analyzed PSD95, a postsynaptic density protein, and BDNF, a neurotrophin essential for synaptic plasticity, both typically reduced in HD models [34, 43, 64, 66]. Western blot analysis of hippocampal lysates from vehicle- and WIN-treated WT and R6/1 mice revealed that WIN administration restored the protein levels of both PSD95 and BDNF in R6/1 mice (Fig. S8b). These findings suggest that WIN improves cognitive function in HD by enhancing both structural and synaptic plasticity.

### Sub-chronic administration of WIN alleviates the impairment of CB1R-mediated presynaptic inhibition in R6/1 mice

Retrograde signaling is a key mechanism through which eCBs modulate synaptic plasticity [67]. Therefore, we next assessed functional changes in CB1R-mediated short-term plasticity at hippocampal synapses in R6/1 mice. Whole-cell patch-clamp recordings were performed on CA1 pyramidal neurons, and both inhibitory and excitatory synaptic transmission were monitored before and after neuronal depolarization in vehicle- and WIN-treated WT and R6/1 mice (Fig. 4c).

In WT hippocampal neurons, a 5-s depolarizing step (from –70 to 0 mV) induced a robust suppression of IPSC amplitude (50.44% ± 5.28%; *n* = 13; *P* < 0.001; paired *t*-test; Fig. 4d). In contrast, R6/1 hippocampal neurons exhibited a significantly reduced DSI compared to WT neurons (29.67% ± 3.39%; *n* = 18; *P* < 0.001, two-way ANOVA, Bonferroni post-hoc test, Fig. 4d). However, this deficit was normalized to WT levels following WIN treatment (52.70% ± 5.65%; *n* = 17; *P* < 0.001; two-way ANOVA, Bonferroni post-hoc test; Fig. 4d). Notably, eCB-induced short-term synaptic plasticity in excitatory terminals, as assessed by DSE, was not altered in R6/1 mice (Fig. S9). Both genotypes exhibited similar depression after neuronal depolarization (*n* = 12 WT vs. *n* = 14 R6/1; *P* > 0.05; Two-way ANOVA, Bonferroni post-hoc test), regardless of WIN treatment (*P* > 0.05; Two-way ANOVA, Bonferroni post-hoc test) (Fig. S9).

#### CB1R protein levels are selectively reduced in hippocampal GABAergic neurons of R6/1 mice

**Fig. 5:**
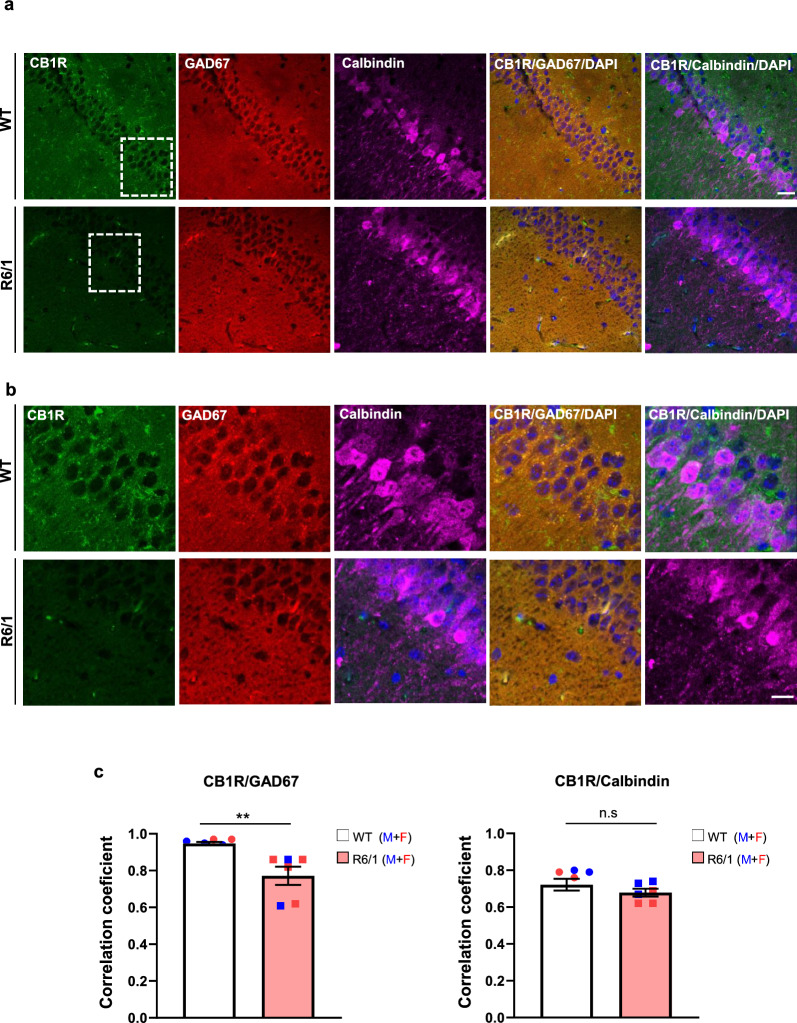
CB1R expression is selectively reduced in GABAergic hippocampal neurons of R6/1 mice. **a** Representative confocal images showing the distribution of CB1R (green), GAD67 (red) and calbindin (purple) in coronal hippocampal sections from 13-week-old naive WT and R6/1 mice. Colocalization of CB1R with GAD67 (GABAergic marker) and CB1R with calbindin (glutamatergic marker) is indicated. Scale bar, 25 µm. **b** High-magnification confocal images of the rectangle areas in **a**, illustrating detailed colocalization of CB1R (green) with GAD67 (red) and with calbindin (purple) in the hippocampus. Scale bar, 10 µm. **c** Quantification of CB1R colocalization with GAD67 and calbindin across the hippocampus, expressed as Pearson’s correlation coefficient. Mean ± SEM, *n* = 6–7 mice per genotype. Student’s *t* test; **P < 0.01

The reduction in hippocampal CB1R levels, along with preserved DSE but impaired DSI in R6/1 mice, suggests differential expression of CB1R among excitatory and inhibitory hippocampal neurons. To investigate this further, double fluorescence staining for CB1R and either calbindin (a marker for excitatory pyramidal neurons) or GAD67 (a marker for inhibitory interneurons) was performed on hippocampal slices from naive WT and R6/1 mice. The immunoreactivity was similar between genotypes and cell markers, suggesting no major differences in the populations of excitatory and inhibitory neurons between R6/1 and WT hippocampi (Fig. S10). Next, the distribution of CB1R in GAD67-positive and calbindin-positive neurons was analyzed (Fig. 5). Double-immunolabeling quantification revealed a strong positive correlation between GAD67 and CB1R, with correlation coefficients ranging from 0.75 to 0.95. However, the coefficient was significantly reduced in R6/1 mice compared to WT mice (Fig. 5b). When analyzing calbindin and CB1R colocalization, lower correlation coefficients (ranging from 0.65 to 0.7) were obtained, consistent with the predominant expression of CB1R in inhibitory hippocampal neurons [36]. Notably, no significant differences were observed between WT and R6/1 mice in this analysis, suggesting the reduction in CB1R level was specific in hippocampal GABAergic neurons of R6/1 mice.

#### Selective CB1R restoration in hippocampal GABAergic neurons improves memory performance of R6/1 mice

**Fig. 6:**
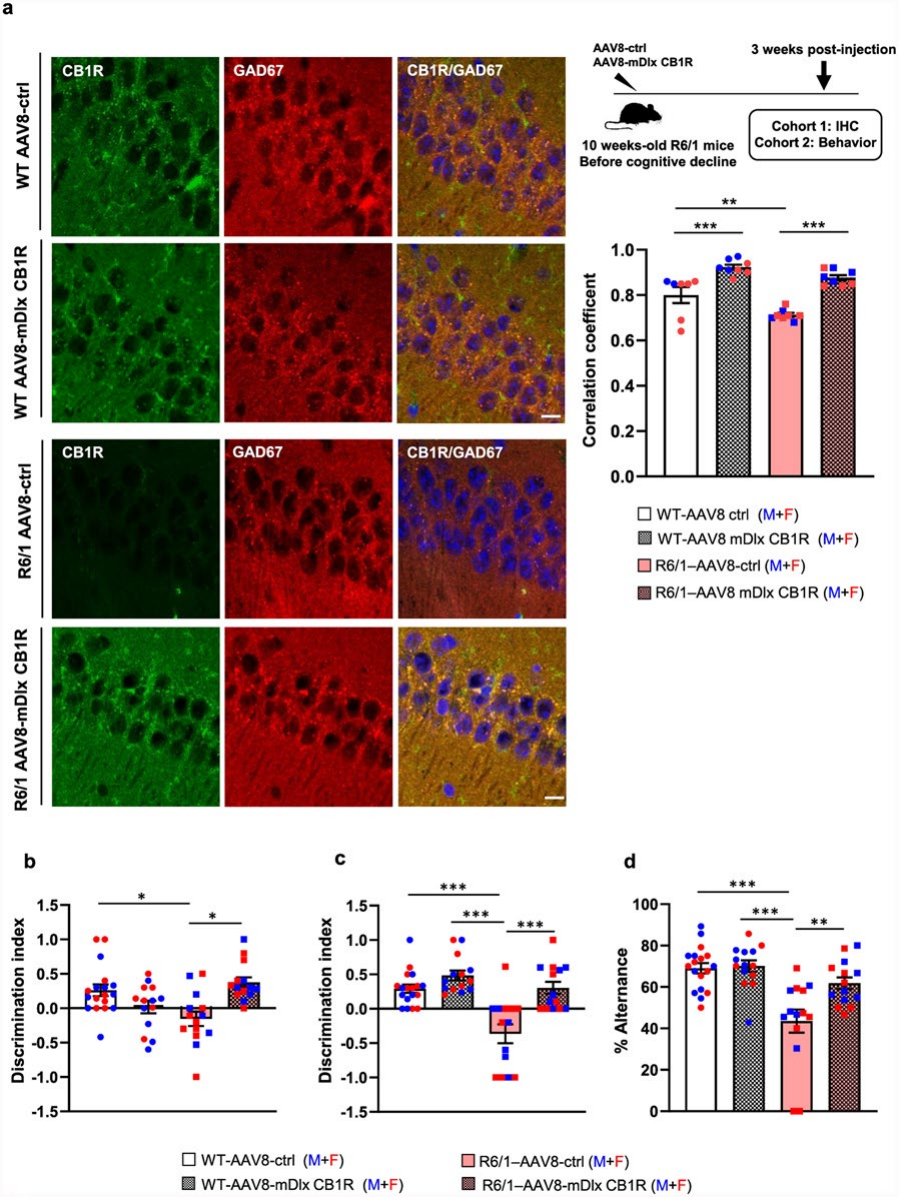
Intrahippocampal infusion of AAV-mDlx-CB1R enhances CB1R expression in GABAergic hippocampal neurons and improves cognitive performance in R6/1 mice. **a** Upper right, schematic illustration of experimental design. WT and R6/1 mice were bilaterally injected in the hippocampus with either AAV8 expressing CB1R under the GABAergic-specific *mDlx* promoter (AAV8-mDlx CB1R) or a control vector expressing mCherry (AAV8-ctrl). Left, representative confocal images show colocalization of CB1R (green) with GAD67 (red) in the hippocampus of transduced WT and R6/1 mice. Scale bar, 10 µm. Right, quantification of CB1R/GAD67 colocalization across the hippocampus, indicated by Pearson’s correlation coefficient. Mean ± SEM, *n* = 7–8 mice per group and genotype. Two-way ANOVA revealed a significant effect of AAV-mDlx-CB1R transduction (*F*(1, 27) = 6.459, *P* = 0.0171) and genotype (*F*(1, 27) = 19.31, *P* = 0.0002). **b** Performance in the novel object location task, assessing spatial memory. Two-way ANOVA revealed a significant interaction effect (*F*(1, 58) = 10.83, *P* = 0.0017). **c** Performance in the novel object recognition task. Two-way ANOVA showed significant effects of AAVs transduction (*F*(1, 50) = 19.03, *P* < 0.0001) and genotype (*F*(1, 50) = 16.28 *P* = 0.0002). **d** Performance in the Y-maze task, assessing spatial working memory. Two-way ANOVA revealed significant effects of AAV transduction (*F*(1, 62) = 8.541, *P* = 0.0048) and genotype (*F*(1, 62) = 28.44, *P* < 0.0001). Mean ± SEM, *n* = 9–18 mice per group and genotype. Statistical comparisons were performed using Tukey's post hoc test, *P < 0.05, **P < 0.01 and ***P < 0.001

The specific reduction of CB1R expression in hippocampal GABAergic neurons led us to hypothesize that restoring CB1R levels in this neuronal population may ameliorate memory deficits. To test this, 10-week-old WT and R6/1 mice received bilateral infusions of either AAV8-mDlx5/6-CB1R (mDlx), which drives CB1R expression selectively in GABAergic neurons, or a control vector (AAV8-mCherry) into the dorsal hippocampus (Fig. S11). One month later, spatial, recognition and working memory were evaluated. We first confirmed the efficiency of AAV8-mDlx5/6-CB1R by assessing CB1R expression in GAD67-positive neurons (Fig. 6a). Consistent with our previous findings (Fig. 5), a significantly lower correlation coefficient between CB1R and GAD67 expression was observed in the hippocampus of the R6/1 mice infused with AAV8-control compared to WT controls (0.7 versus 0.8, *P* < 0.01). Notably, AAV8-mDlx5/6-CB1R infusion significantly increased the correlation coefficient in both genotypes, removing differences between WT and R6/1 mice.

Behaviorally, R6/1 mice transduced with AAV8-mDlx5/6-CB1R exhibited improved performance in the NOLT (Fig. 6b), NORT (Fig. 6c), and Y-maze tests (Fig. 6d), with discrimination indices and alternation percentages comparable to WT mice. These results suggest that CB1R expression in hippocampal GABAergic neurons is crucial for memory function and that its selective restoration can rescue cognitive deficits in R6/1 mice.

## Discussion

Dysregulation of the eCBS, particularly within the basal ganglia, has been observed in both experimental models and patients with HD. This hypofunction, characterized by reduced CB1R signaling, has been linked to striatal neurodegeneration, dysfunction, and the motor disturbances commonly associated with HD [4–6, 10, 11]. However, most of these studies have predominantly focused on motor symptoms, overlooking the broader clinical spectrum of HD. Notably, cognitive impairments and behavioral disturbances often manifest prior to motor decline and significantly contribute to the overall functional decline observed in HD patients. Given the central role of the hippocampus in learning and memory as well as its sensitivity to eCBS signaling, it is plausible that dysregulation of the eCBS within the hippocampus contributes to early cognitive deficits in HD. This raises an important yet unexplored question: does eCBS dysfunction in the hippocampus underlie the memory decline observed in HD? In this study, we employed a combination of immunohistological, biochemical, electrophysiological, pharmacological, and genetic approaches to provide strong experimental evidence for the specific contribution of GABAergic CB1R dysfunction to the cognitive impairments observed in HD. Our data demonstrate that in the presence of mHtt expression, both the level and the affinity of hippocampal CB1Rs are reduced, while CB2R expression remains unchanged. Notably, pharmacological activation of the remaining CB1Rs alleviates memory dysfunction in HD models.

Moreover, CB1R activation restored DSI in the HD hippocampus, suggesting that disrupted excitatory–inhibitory balance is a key mechanism underlying cognitive decline in this disease. Accordingly, selective expression of CB1Rs in hippocampal GABAergic interneurons alone was sufficient to ameliorate memory deficits, underscoring the critical contribution of GABAergic CB1R dysfunction to the cognitive symptoms of HD.

Several studies have reported that the eCBS activity plays a critical role in modulating cognitive processes [34, 63, 68]. Under physiological conditions, activation of CB1R appears to exert a detrimental effect on memory [63, 69]. Conversely, a reduction in CB1R expression, such as that observed during aging or in neurodegenerative disorders, is associated with cognitive impairments [70–72]. In line with these findings, we detected a significant and specific reduction of CB1R protein levels in the hippocampus of both HD patients and HD mouse models. Interestingly, CB1R mRNA expression in the hippocampus of HD mice remained unchanged, suggesting that the observed reduction of protein level is likely due to post-transcriptional mechanisms rather than altered gene expression.

Notably, this CB1R reduction was concomitant with impaired performance in cognitive tasks evaluating spatial, recognition and working memory. Similar cognitive deficits have been reported in CB1R*-*null mice, including impairments in both recognition and spatial memory [73–75]. Furthermore, genetic deletion of CB1R in Alzheimer’s disease (AD) mouse models exacerbates disease pathology and accelerates cognitive decline [72, 76]. Further supporting the involvement of CB1R hypofunction in HD-related cognitive deficits, we demonstrated that sub-chronic administration of the CB1R agonist WIN effectively ameliorated memory impairments in HD mice. Notably, these beneficial effects were observed both with treatment initiated at presymptomatic stages, prior to the onset of detectable cognitive decline, and at symptomatic stages, when memory impairments are already evident [29, 30].

To gain insights into the molecular mechanisms underlying the memory improvements observed with CB1R activation, we first examined changes in CB1R expression. Previous studies have shown that treatment with CB1R-selective positive allosteric modulators can enhance motor coordination and restore CB1R levels in the striatum and cortex of HD mouse models [77]. Additionally, low-dose administration of WIN has been shown to increase CB1R activity in the cortex of a rat model of cholinergic degeneration, resulting in improved cognitive performance [78], whereas in a rat model of cerebral ischemia, WIN treatment significantly increased CB1R protein levels in the hippocampus [79]. Consistent with these findings, our immunohistochemical and Western blot analyses revealed a recovery of CB1R protein level in the hippocampus of two different HD mouse models (R6/1 and Hdh ^Q7/Q111^ mice) following sub-chronic WIN treatment. Additionally, radioligand binding assays showed higher B_max_ values in the hippocampus of WIN-treated R6/1 mice, suggesting an increase in receptor density. WIN treatment also modulated CB1R affinity states. In WT mice, CB1Rs exhibited two distinct active conformations corresponding to high- and low-affinity states. In contrast, untreated R6/1 mice exhibited only the high-affinity state, though at reduced levels compared to WT mice. Notably, WIN treatment reestablished both affinity states in R6/1 mice. These findings suggest that the WIN-induced overactivation of the remaining CB1Rs in the hippocampus of R6/1 mice promotes a shift toward an increased proportion of receptors in the high-affinity state. This shift may enhance the overall CB1R signaling capacity and contribute to the observed improvements in memory function in HD models.

To evaluate the functional relevance of CB1R restoration following WIN treatment, we examined several pathophysiological hallmarks of HD. One of the most prominent features of HD is the accumulation of mHtt aggregates, which are believed to contribute to cellular dysfunction and neurodegeneration [80]. Accordingly, considerable efforts have been directed toward developing small molecules that promote the clearance and degradation of mHtt aggregates [81, 82]. In our study, WIN treatment significantly reduced the number of mHtt inclusions in the hippocampus of R6/1 mice, suggesting a modulatory role of CB1R signaling in mHtt protein aggregation. This is consistent with previous studies showing that genetic deletion of CB1R exacerbates striatal huntingtin aggregation in HD mouse models [6, 83], and that pharmacological activation of CB1R with Δ9-tetrahydrocannabinol (THC) alleviates mHtt accumulation [6].

In addition to its impact on protein aggregation, WIN treatment exerted beneficial effects on structural and synaptic plasticity. Specifically, it normalized the expression of synaptic markers such as PSD95 and the neurotrophin BDNF in the hippocampus of R6/1 mice and improved dendritic spine density in the CA1 region. The interaction between CB1R signaling, BDNF expression, and dendritic spines is well-documented. For instance, reduced hippocampal BDNF levels have been observed in CB1R-deficient mice [84–86], while activation of the BDNF–TrkB pathway has been shown to stabilize and enlarge dendritic spines [87, 88]. Moreover, chronic administration of low-dose THC in aged mice improves cognitive function by increasing cortical spine density and synaptic protein expression [74, 89]. Similarly, chronic WIN treatment in young rats promotes cytoskeletal remodeling in the hippocampus, resulting in increased dendritic thickness and branching [90].

Endocannabinoids, acting as retrograde signaling messengers, are known to also modulate short-term synaptic plasticity [64, 91]. To investigate whether CB1R-mediated short-term plasticity is altered in HD, we assessed both inhibitory (DSI) and excitatory (DSE) synapses [92]. In the hippocampus of R6/1 mice, CB1R-mediated presynaptic inhibition was selectively impaired at inhibitory, but not excitatory, terminals. This reduction in DSI may contribute to an imbalance between excitatory and inhibitory signaling in the HD hippocampus, potentially disrupting cognitive function.

Notably, the reduction of CB1R protein level in R6/1 mice was predominantly observed in hippocampal GABAergic interneurons, likely accounting for the impaired DSI response. This cell-type-specific downregulation is consistent with previous reports in R6/2 mice, where a marked decrease in CB1R was detected specifically in striatal GABAergic neurons, implicating GABAergic circuit dysfunction in striatal excitotoxicity [93, 94]. Moreover, deficits in tonic GABAergic inhibition have been identified in the cortex and striatum across different HD mouse models, often preceding the appearance of overt symptoms. These findings support the hypothesis that therapeutic strategies aimed at restoring GABAergic neurotransmission could be beneficial for HD patients. Consistent with this notion, our findings demonstrate that selective recovery of CB1R expression in hippocampal GABAergic neurons rescued deficits in working, spatial and recognition memory in R6/1 mice. These findings underscore the critical role of GABAergic dysregulation in the hippocampus in mediating the cognitive impairments associated with HD and highlight CB1R signaling as a potential therapeutic target for early cognitive deficits.

However, our results should be interpreted in light of some limitations. First, WIN is not a selective CB1R agonist, and thus we cannot exclude the possibility that the observed beneficial effects may, at least in part, be mediated by CB1R-independent mechanisms. For instance, WIN has been shown to exert neuroprotective effects in models of vascular dementia and ischemic injury, possibly through anti-inflammatory and antioxidative pathways [79, 95]. A second limitation relates with the systemic administration of WIN (i.p. injection), which raises the question of whether CB1R activation in other brain regions could also influence cognitive improvements. Indeed, other types of memory, such as fear memory and social cognition, both of which are affected in HD patients [96–99], could potentially be impacted by our pharmacological approach. Therefore, future studies aimed at exploring these aspects are needed.

## Conclusion

In summary, we propose that the selective loss of CB1R in hippocampal GABAergic neurons disrupts the CB1R-mediated short-term synaptic plasticity, thereby contributing to memory decline in HD. Therapeutic strategies aimed at restoring inhibitory GABAergic function through CB1R modulation may offer a promising approach to alleviate cognitive dysfunction in HD patients.

## Supplementary Information

**Additional file 1**. **Figure S1**. CB1R mRNA expression in the hippocampus of R6/1 mice remains unchanged across disease progression. **Figure S2**. CB1R protein levels are not altered in the cerebellum of R6/1 mice across disease progression. **Figure S3**. Behavioral effects of WIN-55212-2 treatment in WT mice. **Figure S4**. Sub-chronic WIN-55212-2 treatment in R6/1 mice at presymptomatic stages prevents memory deficits. **Figure S5**. Sub-chronic WIN-55212-2 treatment improves memory performance and restores hippocampal CB1R expression in symptomatic Hdh^Q7/Q111 ^mice. **Figure S6**. Sub-chronic WIN-55212-2 treatment restores CB1R binding affinity. **Figure S7**. Hippocampal CB2R expression is unaltered in naive or WIN-55212-2-treated HD mice. **Figure S8**. Sub-chronic WIN-55212-2 treatment recovers the expression of synaptic-related proteins in R6/1 mice. **Figure S9**. Depolarization-induced suppression of excitation (DSE) is not altered in the hippocampus of R6/1 mice. **Figure S10**. Characterization of calbindin and GAD67 expression in the hippocampus of WT and R6/1 mice. **Figure S11**. Characterization of AAV8-mCherry transduction in the hippocampus of WT and R6/1 mice.

**Additional file** **2**. Uncropped Western blots.

## Data Availability

All data generated or analyzed during this study are included in this article and Additional file or are available from the corresponding author on reasonable request. This study did not generate new unique reagents.

## Abbreviations

aCSF: Artificial cerebrospinal fluid
BDNF: Brain derived neurotrophic factor
CB1R: Cannabinoid receptor type I
DAPI: 4',6-Diamidino-2-phenylindole
DG: Dentate gyrus
DSE: Depolarization-induced suppression of excitation
DSI: Depolarization-induced suppression of inhibition
eCBS: Endocannabinoids
ECL: Enhanced chemiluminescent
EPSCs: Excitatory postsynaptic currents
GAD67: Glutamate decarboxylase 67
HD: Huntington´s disease
HEPEs: 2-(4-(2-Hydroxyethyl)-1-piperazinyl)-ethanesulfonic acid
IPSCs: Inhibitory postsynaptic currents
NOLT: Novel object location task
NORT: Novel object recognition task
PBS: Phosphate-buffered saline
PSD95: Postsynaptic density protein 95
SDS: Sulfat dodecyl sulfate
SDS-PAGE: Sodium dodecyl sulfate–polyacrylamide gel electrophoresis
THC: Δ9-Tetrahydrocannabinol
