# Supplementary material for: Restoration of CB1 receptor function in hippocampal GABAergic neurons rescues memory deficits in Huntington’s disease models
Source: Transl Neurodegener. 2025 Aug 25;14:44. doi: 10.1186/s40035-025-00500-w (PMC12376758; doi:10.1186/s40035-025-00500-w)
Supplement: Supplementary file 1 — Additional file 1. Figure S1. CB1R mRNA expression in the hippocampus of R6/1 mice remains unchanged across disease progression. Figure S2. CB1R protein levels are not altered in the cerebellum of R6/1 mice across disease progression. Figure S3. Behavioral effects of WIN-55212-2 treatment in WT mice. Figure S4. Sub-chronic WIN-55212-2 treatment in R6/1 mice at presymptomatic stages prevents memory deficits. Figure S5. Sub-chronic WIN-55212-2 treatment improves memory performance and restores hippocampal CB1R expression in symptomatic HdhQ7/Q111 mice. Figure S6. Sub-chronic WIN-55212-2 treatment restores CB1R binding affinity. Figure S7. Hippocampal CB2R expression is unaltered in naive or WIN-55212-2-treated HD mice. Figure S8. Sub-chronic WIN-55212-2 treatment recovers the expression of synaptic-related proteins in R6/1 mice. Figure S9. Depolarization-induced suppression of excitation (DSE) is not altered in the hippocampus of R6/1 mice. Figure S10. Characterization of calbindin and GAD67 expression in the hippocampus of WT and R6/1 mice. Figure S11. Characterization of AAV8-mCherry transduction in the hippocampus of WT and R6/1 mice. [file 40035_2025_500_MOESM1_ESM.pdf]

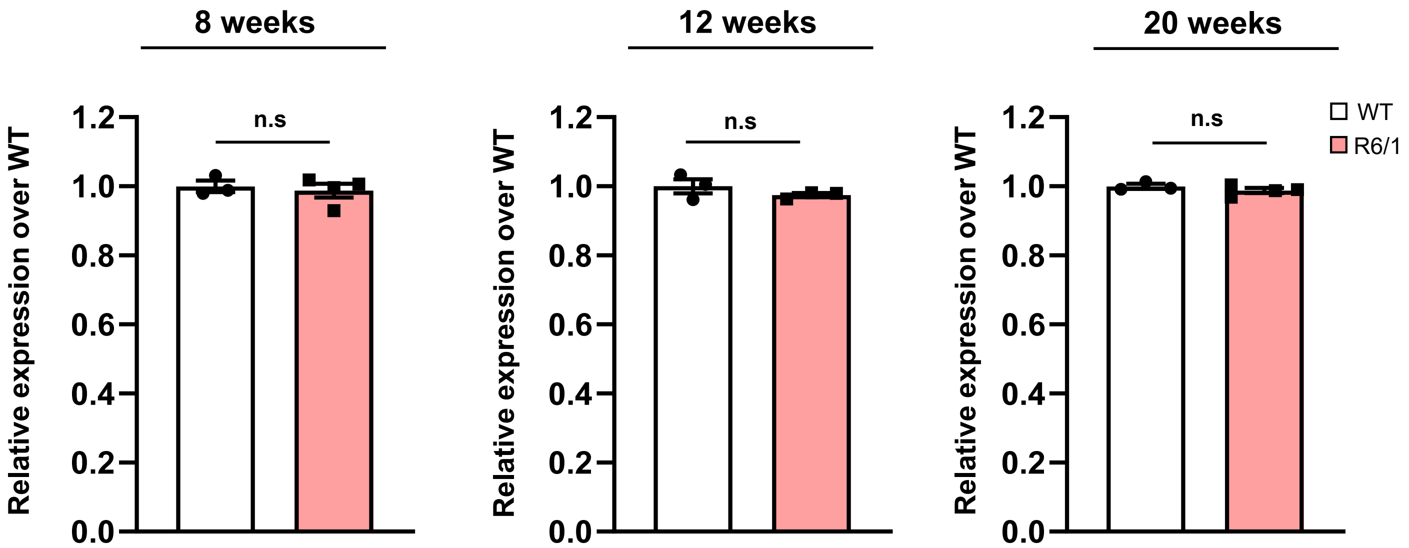

Figure S1. CB1R mRNA expression in the hippocampus of R6/1 mice remains unchanged across disease progression. Quantification of *Cnr1* (CB1R) mRNA expression in the hippocampus of WT and R6/1 mice at different stages of disease progression, measured by quantitative real-time PCR (qRT-PCR). Expression levels are presented as fold change relative to WT controls at each time point. No significant differences in *Cnr1* mRNA expression were detected between genotypes at any age analyzed. Data are presented as mean ± standard error of the mean (SEM) with n=3-4 mice per group. Statistical significance was performed using Mann-Whitney test.

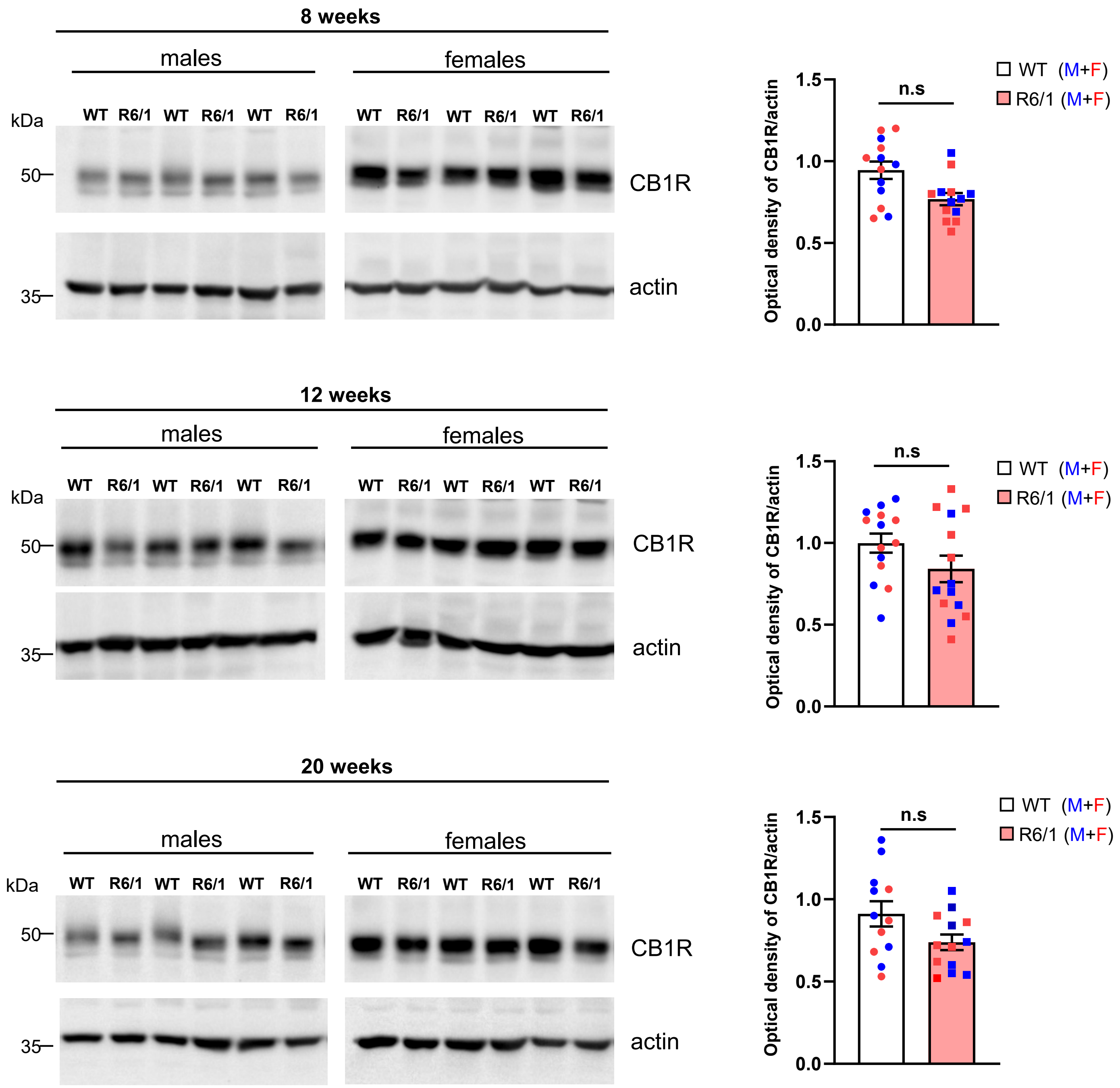

Figure S2. CB1R protein levels are not altered in the cerebellum of R6/1 mice across disease progression. Representative immunoblots showing CB1R protein levels in total cerebellar lysates from WT and R6/1 mice at different stages of disease progression. Actin was used as a loading control. Histograms display CB1R protein levels normalized to actin (optical density). Data are presented as mean ± standard error of the mean (SEM) with n= 13-14 mice per group. Statistical significance was performed using Mann-Whitney test.

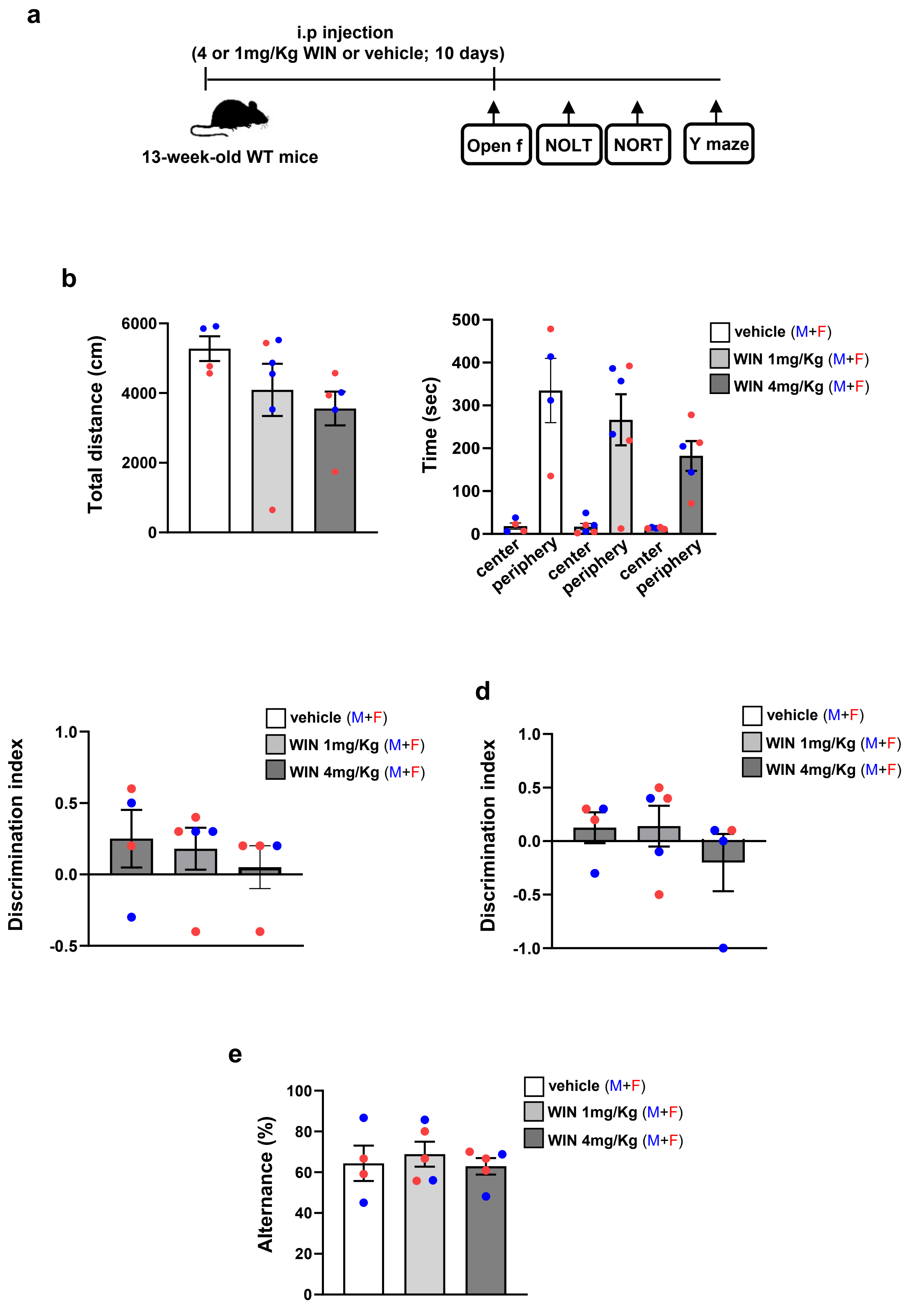

Supplementary Figure 3

Figure S3. Behavioral effects of WIN-55,212-2 treatment in WT mice.

a Schematic timeline showing the WIN administration protocol, followed by the sequence of behavioral tests. **b** Motor coordination and exploratory behavior assessed in the open field test, showing total distance traveled (cm) and time spent in the center versus periphery (seconds). **c** Spatial memory performance evaluated using the Novel Object Location Task (NOLT). **d** Recognition memory assessed with the Novel Object Recognition Task (NORT). **e** Spatial working memory assessed by the Y-maze test. Statistical analysis was performed using one-way ANOVA. Data are presented as mean ± standard error of the mean (SEM) with n=4-6 mice per group.

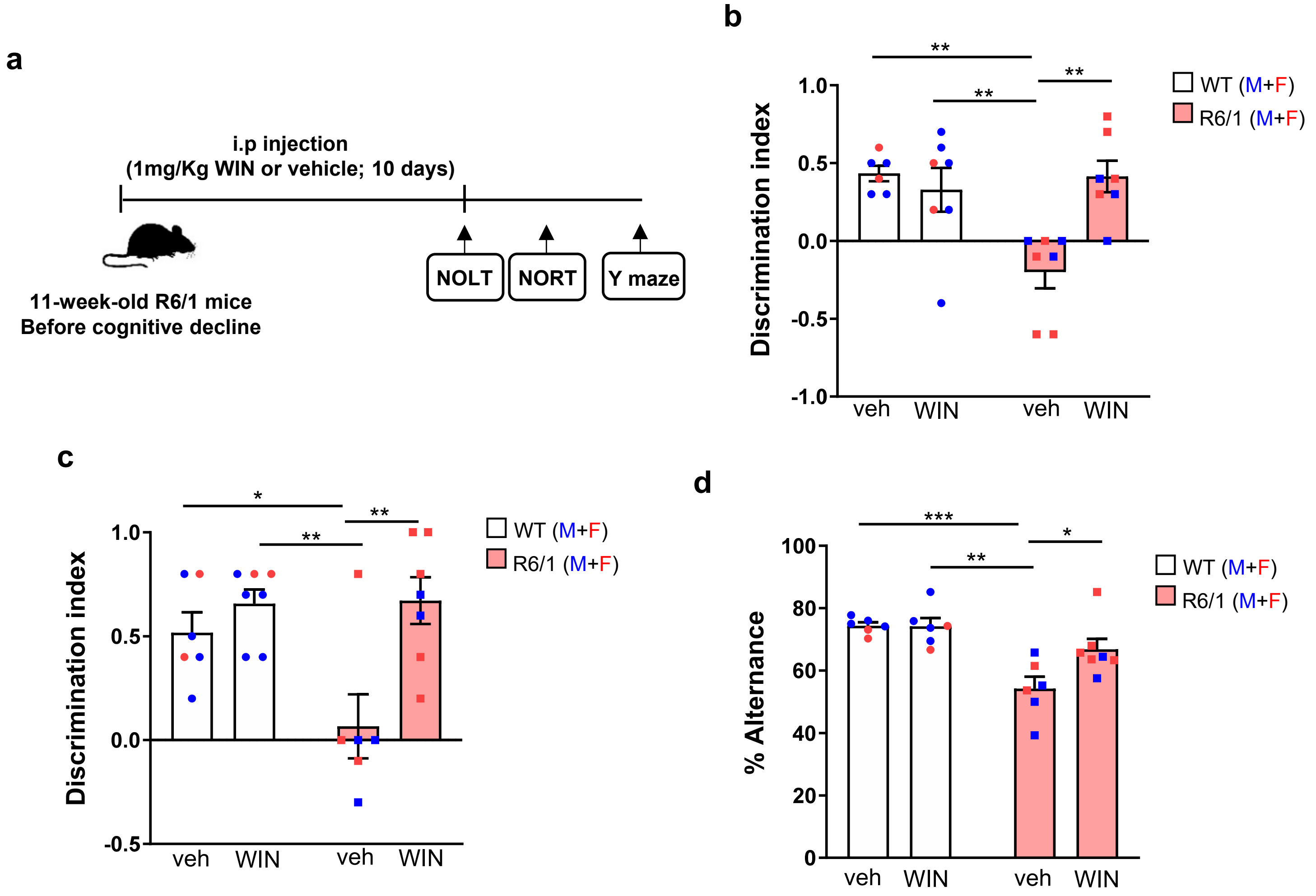

Figure S4. Sub-chronic WIN-55,212-2 treatment in R6/1 mice at presymptomatic stages prevents memory deficits.

a Schematic timeline showing the WIN administration protocol, followed by the sequence of behavioral tests. **b** Performance in the Novel Object Location Task (NOLT), assessing spatial memory. Two-way ANOVA revealed significant effects of WIN treatment ($F(1,36) = 6.785$, $p=0.0133$) and genotype ($F(1, 36)=4.918$, $p=0.033$). **c** Performance in the Novel Object Recognition Task (NORT). Two-way ANOVA revealed a significant effect of WIN treatment ($F(1,22) = 1.39$, $p=0.0027$). **d** Performance in the Y-maze task, assessing working spatial memory. Two-way ANOVA revealed significant effects of WIN treatment ($F(1,21) = 4.479$, $p=0.0464$) and genotype ($F(1, 21) = 22.02$, $p=0.0001$). Data are presented as mean ± standard error of the mean (SEM) with n=8-12 animals per group and genotype. Post hoc comparisons using Tukey's test indicated $*p<0.05$, $**p<0.01$ and $***p<0.001$.

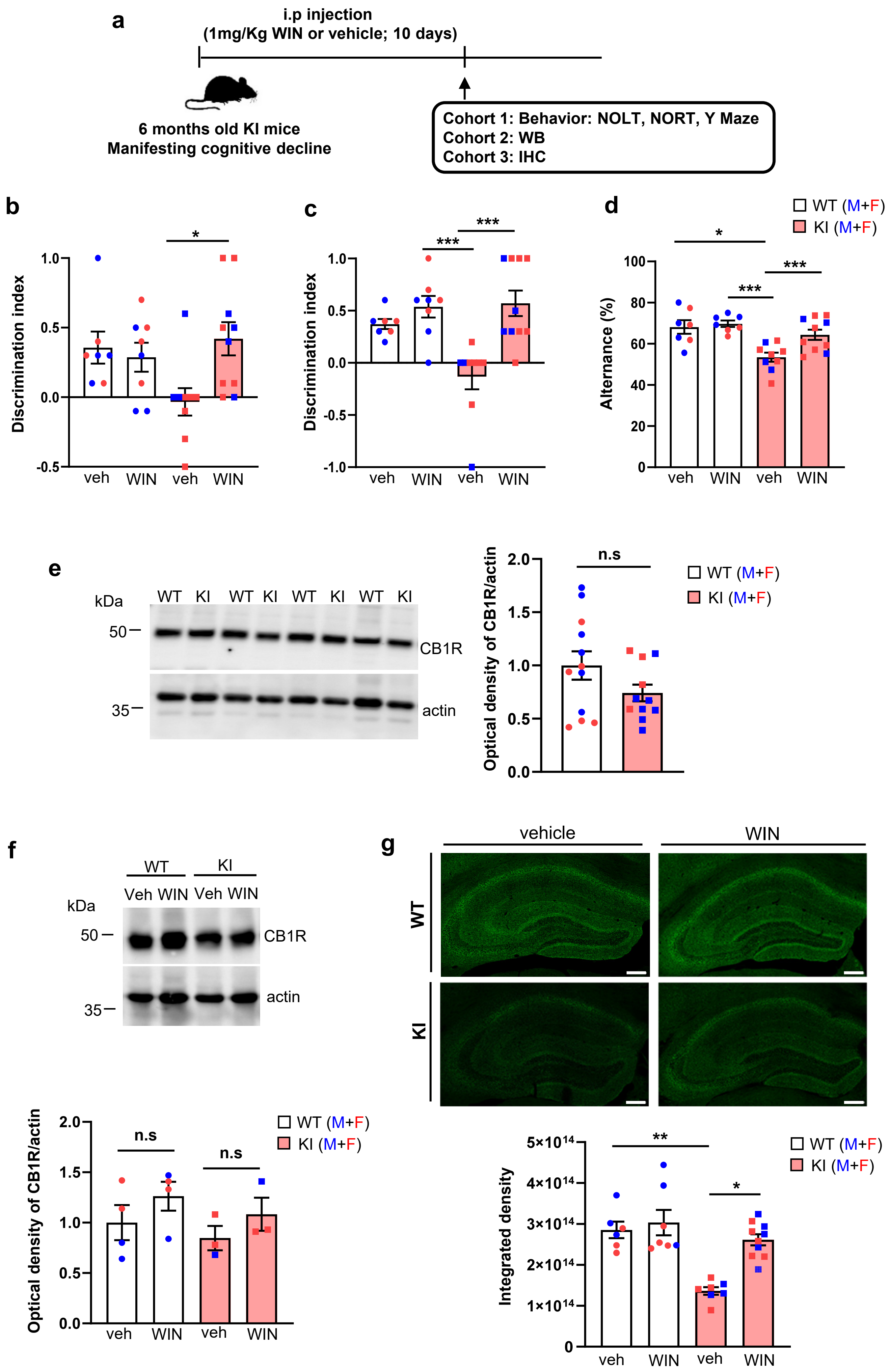

a
i.p injection
(1mg/Kg WIN or vehicle; 10 days)
6 months old KI mice
Manifesting cognitive decline
Cohort 1: Behavior: NOLT, NORT, Y Maze
Cohort 2: WB
Cohort 3: IHC
b
Discrimination index
veh
WIN
veh
WIN
c
Discrimination index
veh
WIN
veh
WIN
d
WT (M+F)
KI (M+F)
Alternance (%)
veh
WIN
veh
WIN
e
kDa
WT KI WT KI WT KI WT KI
50
35
CB1R
actin
Optical density of CB1R/actin
n.s
WT (M+F)
KI (M+F)
f
WT
KI
Veh WIN Veh WIN
kDa
50
35
CB1R
actin
g
vehicle
WIN
WT
KI
Optical density of CB1R/actin
n.s
n.s
WT (M+F)
KI (M+F)
veh
WIN
veh
WIN
Integrated density
WT (M+F)
KI (M+F)
veh
WIN
veh
WIN

Figure S5. Sub-chronic WIN-55,212-2 treatment improves memory performance and restores hippocampal CB1R expression in symptomatic Hdh$^{Q7/Q111}$mice.

a Schematic timeline showing the WIN administration protocol, followed by the sequence of behavioral tests and biochemical analyses. **b** Spatial memory performance assessed using the Novel Object Location Task (NOLT). Two-way ANOVA revealed significant effects of WIN treatment (F(1,62)=8.541, p=0.0048) and genotype (F(1,62)=28.44 p<0.0001). **c.** Recognition memory assessed using the Novel Object Recognition Task (NORT). Two-way ANOVA revealed significant effects of WIN treatment (F(1,30) =15.30, p=0.0005) and genotype (F(1,30) =4.515 p=0.0420). **d.** Spatial working memory performance assessed using the Y-maze test. Two-way ANOVA revealed significant effects of WIN treatment (F(1,29) =6.161, p=0.0191) and genotype (F(1,29) =15.93, p=0.0004). Data are presented as mean ± standard error of the mean (SEM) with n=6-10 mice per group. Tukey´s pots hoc test indicated *p<0.05 and ***p<0.001. **e.** Representative immunoblots showing CB1R protein levels in total hippocampal lysates from naive Hdh$^{Q7/Q7}$ (WT) and Hdh$^{Q7/Q111}$ (KI) mice at 6 months of age. Actin was used as a loading control. Histogram shows CB1R levels normalized to actin (optical density). Data are presented as mean ± standard error of the mean (SEM) with n= 11-12 mice per group. Statistical significance was performed using Mann-Whitney test. **f.** Representative immunoblots of CB1R in hippocampal lysates from vehicle-and-WIN-treated Hdh$^{Q7/Q7}$ (WT) and Hdh$^{Q7/Q111}$ (KI) mice. Actin was used as a loading control. Histogram shows CB1R levels normalized to actin (optical density). Data are presented as mean ± standard error of the mean (SEM) with n= 3-4 mice per group and genotype. **g.** Representative confocal images showing CB1R immunofluorescence in hippocampal coronal sections from vehicle or WIN-treated Hdh$^{Q7/Q7}$ (WT) and Hdh$^{Q7/Q111}$ (KI) mice. Scale bar = 100 μm. Data are presented as the mean ± standard error of the mean (SEM) with n = 6–10 mice per group and genotype. Two-way ANOVA revealed significant effects of WIN treatment (F(1,30) =12.05, p=0.0016) and genotype (F(1,30) =8.322 p=0.0072). Tukey´s pots hoc test indicated *p<0.05 and **p<0.01.

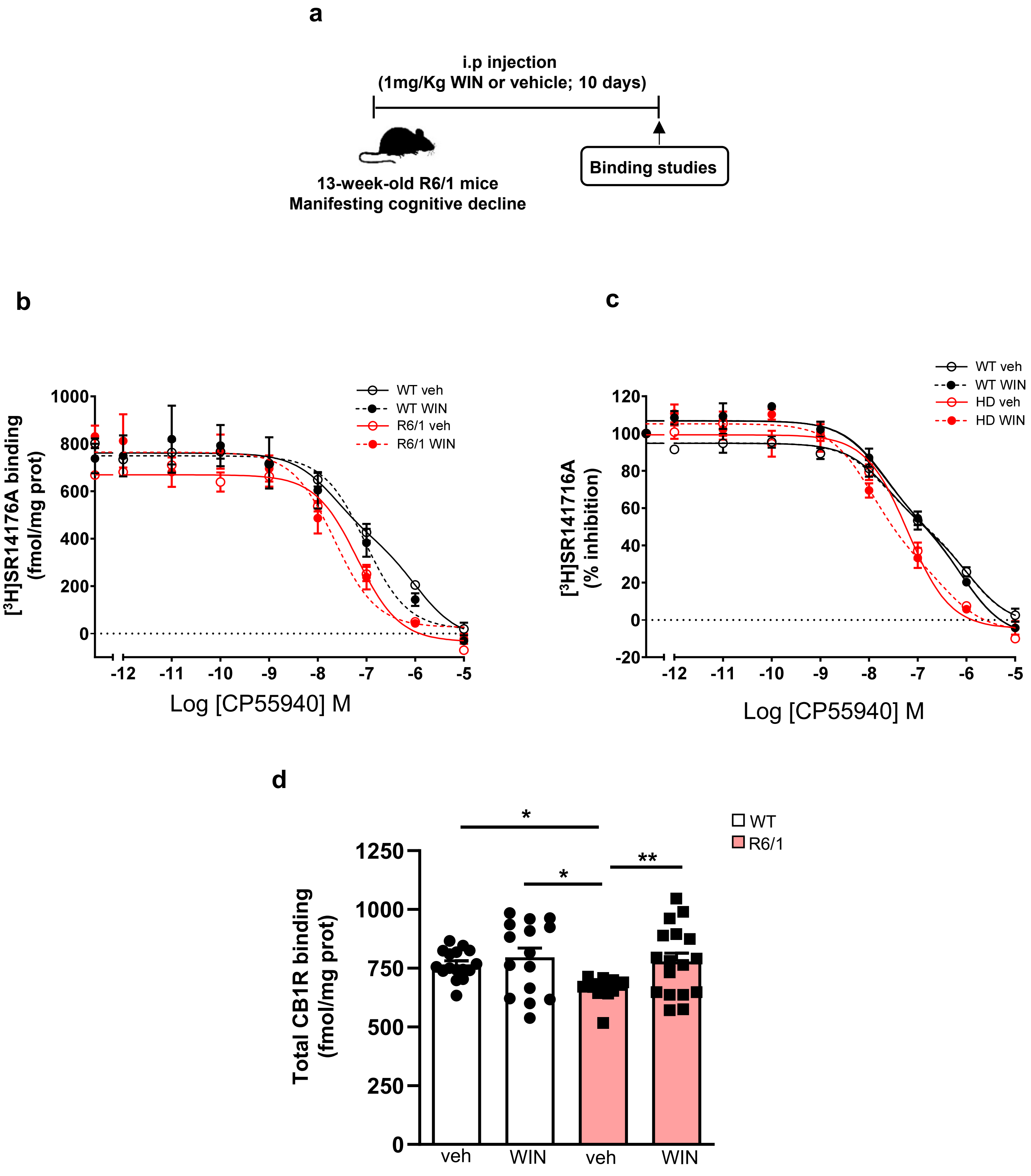

a
i.p injection
(1mg/Kg WIN or vehicle; 10 days)
13-week-old R6/1 mice
Manifesting cognitive decline
Binding studies
b
[3H]SR14176A binding
(fmol/mg prot)
1000
800
600
400
200
0
-12
-11
-10
-9
-8
-7
-6
-5
Log [CP55940] M
WT veh
WT WIN
R6/1 veh
R6/1 WIN
c
[3H]SR141716A
(% inhibition)
120
100
80
60
40
20
0
-20
-12
-11
-10
-9
-8
-7
-6
-5
Log [CP55940] M
WT veh
WT WIN
HD veh
HD WIN
d
Total CB1R binding
(fmol/mg prot)
1250
1000
750
500
250
0
veh
WIN
veh
WIN
*
*
**
WT
R6/1

Figure S6. Sub-chronic WIN-55,212-2 treatment restores CB1R binding affinity.
a Schematic timeline showing the WIN administration protocol, followed by the CB1R binding analysis. **b** [^{3}H] SR141716A binding assays inhibition curves by CP55,940 (fmol/mg protein) in hippocampal membrane homogenates from vehicle-treated WT mice (n = 14), WIN-treated WT mice (n = 10), vehicle-treated R6/1 mice (n = 10) and WIN-treated R6/1 mice (n = 9). Statistical analysis using an F-test revealed significant differences between groups ($p< 0.001$). **c** Percentage of [^{3}H]SR141716A binding inhibited by CP55,940. Significant differences in IC50 values and best-fit models (one or two binding sites) were observed between groups, with an F-test indicating statistical significance ($p< 0.001$). d. Total binding of [3H]SR141716A, measured under conditions corresponding to the maximum binding (Bmax), reflects CB1R density and is expressed as fmol/mg of protein. Data are presented as mean $\pm$ standard error of the mean (SEM) (n= 4-6 mice per group and genotype). Two-way ANOVA revealed a significant effect of WIN treatment ($F(1,60)=7.188$, $p=0.0095$) and genotype ($F(1,60)=5.313$, $p=0.0246$). Post hoc comparisons using Tukey's test indicated *$p<0.05$, **$p<0.01$.

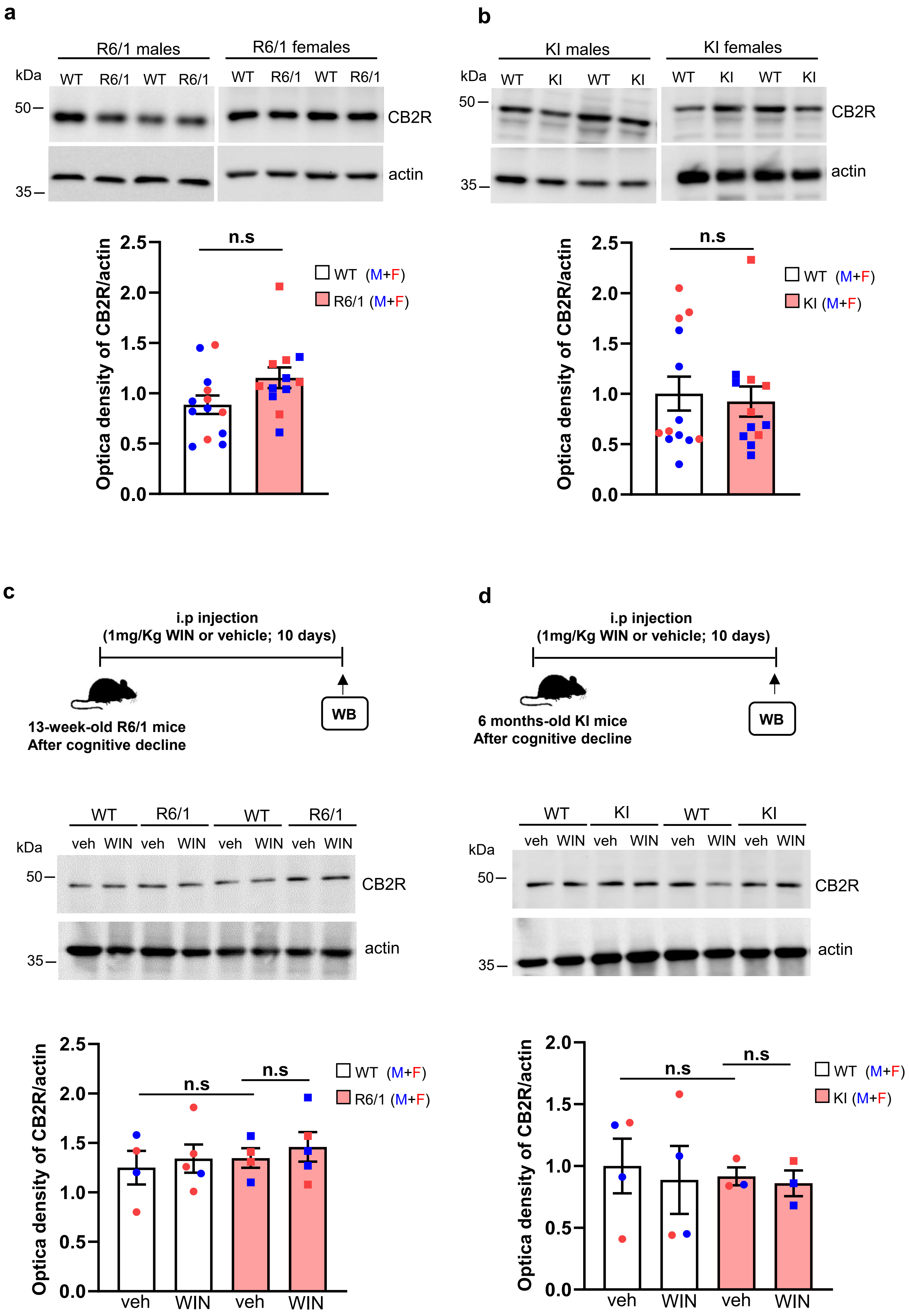
a
R6/1 males
R6/1 females
kDa
WT
R6/1
50
35
CB2R
actin
n.s
Optica density of CB2R/actin
WT (M+F)
R6/1 (M+F)
b
KI males
KI females
KI
KI (M+F)
c
i.p injection
(1mg/Kg WIN or vehicle; 10 days)
13-week-old R6/1 mice
After cognitive decline
WB
veh
WIN
d
6 months-old KI mice
After cognitive decline

Figure S7. Hippocampal CB2R expression is unaltered in naive or WIN-55,212-2-treated HD mice.
Representative immunoblots showing CB2R levels in total hippocampal lysates from naive WT and R6/1 mice at 12 weeks of age (**a**) and $Hdh^{Q7/Q7}$ (WT) and $Hdh^{Q7/Q111}$(KI) mice at 6 months of age (**b**). Actin was used as a loading control. Histograms show CB2R protein levels normalized to actin (optical density). Data are presented as the mean ± standard error of the mean (SEM) with n= 12-13 mice per group. Statistical significance was determined by the Mann-Whitney test. **c and d.** Schematic timeline showing the WIN administration protocol, followed by the subsequent biochemical assays. Representative immunoblots showing CB2R levels in total hippocampal lysates from vehicle and WIN-treated WT and R6/1 mice (**c**) and $Hdh^{Q7/Q7}$ (WT) and $Hdh^{Q7/Q111}$ (KI) mice (**d**). Actin was used as a loading control. Histograms show CB2R protein levels normalized to actin (optical density). Data are presented as the mean ± standard error of the mean (SEM) with n= 3-4 mice per group and genotype. Statistical significance was assessed by Mann-Whitney test.

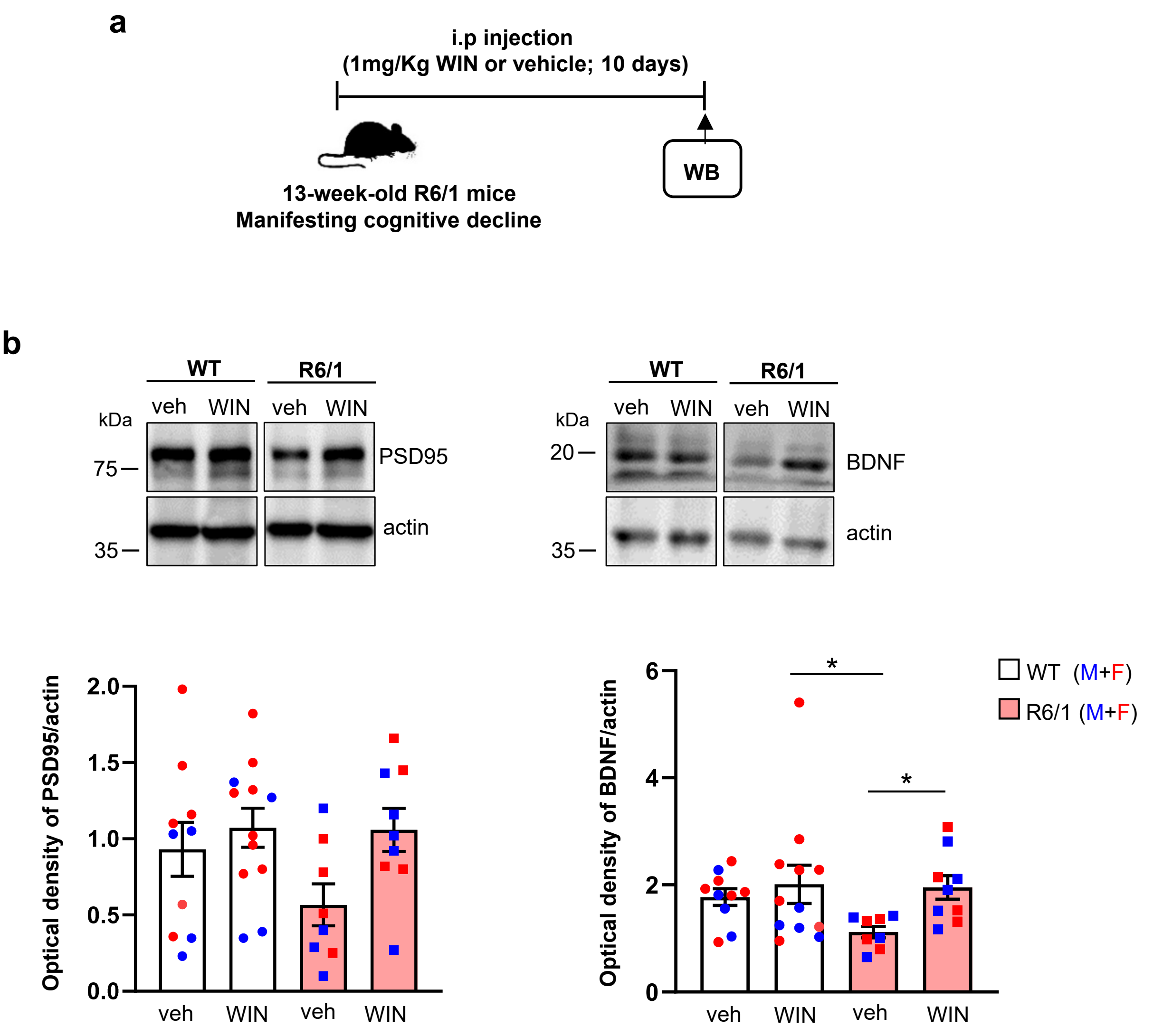

Figure S8. Sub-chronic WIN-55,212-2 treatment recovers the expression of synaptic-related proteins in R6/1 mice.

a Schematic timeline showing the WIN administration protocol, followed by the sequence of biochemical analyses. **b** Representative immunoblots showing the levels of PSD95 and BDNF in total hippocampal lysates from vehicle-and-WIN-treated WT and R6/1 mice. Actin was used as a loading control. Histograms show PSD95 and BDNF protein levels normalized to actin (optical density). Data are presented as the mean ± standard error of the mean (SEM) with n = 8-12 mice per group. Statistical analysis was performed using two-way ANOVA, revealing significant effects of WIN treatment on PSD95 (WIN effect $F(1,33) = 4.481$, p=0.04) and BDNF (WIN effect $F(1,33)=6.568$, p=0.0151). Tukey´s pots hoc test indicated *$p < 0.05$.

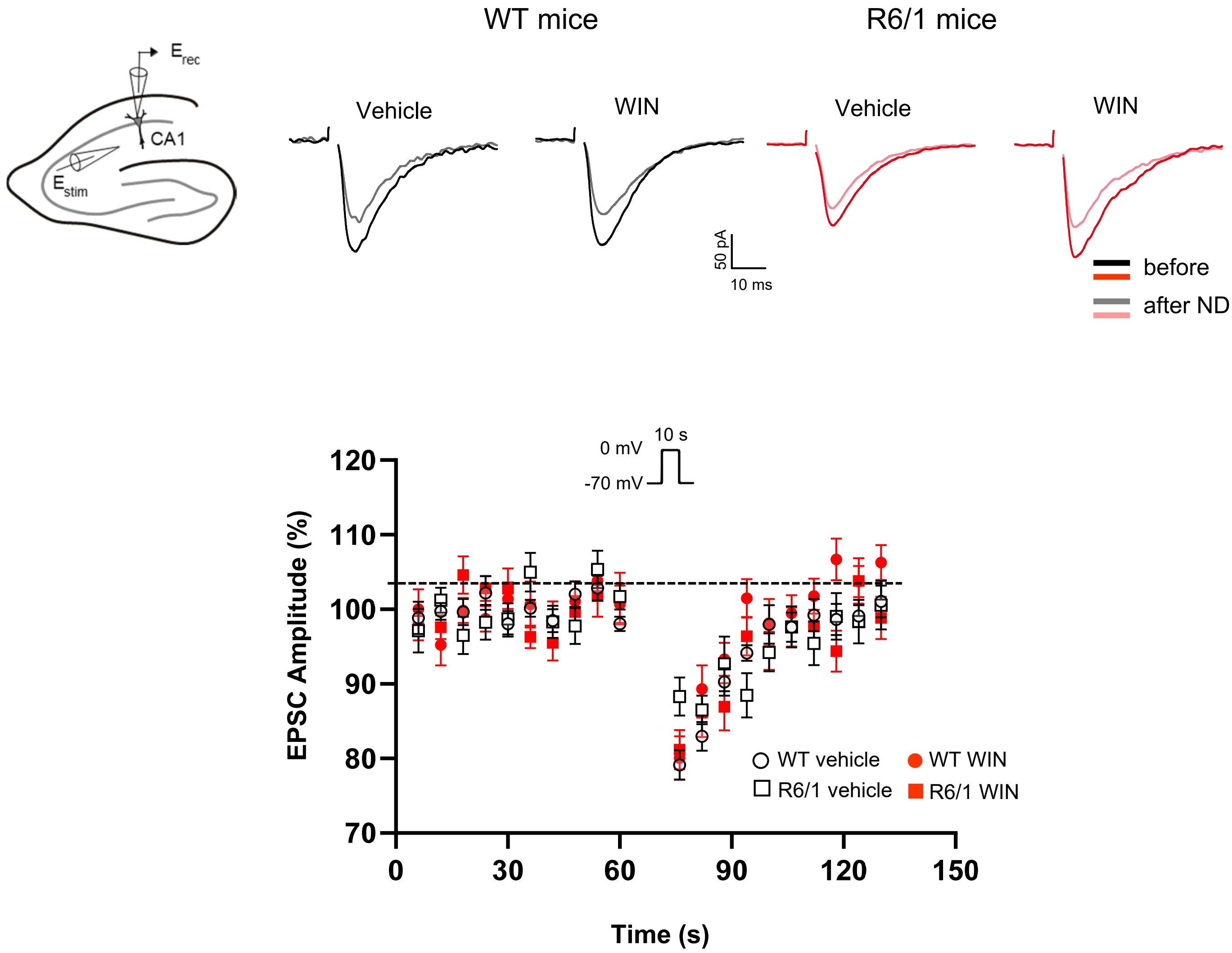

Figure S9. Depolarization-induced suppression of excitation (DSE) is not altered in the hippocampus of R6/1 mice.

Representative traces of excitatory postsynpatic currents (EPSCs), averaged from two responses, recorded from CA1 pyramidal neurons before (pre) and after (post) a 10-second depolarizing step to 0 mV, ilustrating DSE under different experimental conditions. Normalized average EPSC amplitude before and after depolarization are shown for the following experimental conditions: vehicle-treated WT (n=12 neurons, 6 mice), vehicle-treated R6/1 (n= 14 neurons, 6 mice), WIN-treated (n= 10 neurons, 4 mice), and WIN-treated R6/1 (n= 20 neurons, 5 mice). Data are presented as mean ± standard error of the mean (SEM). Statistical significance was determined using two-way ANOVA with Bonferroni post-hoc test. Both male and female mice were included in all analysis.

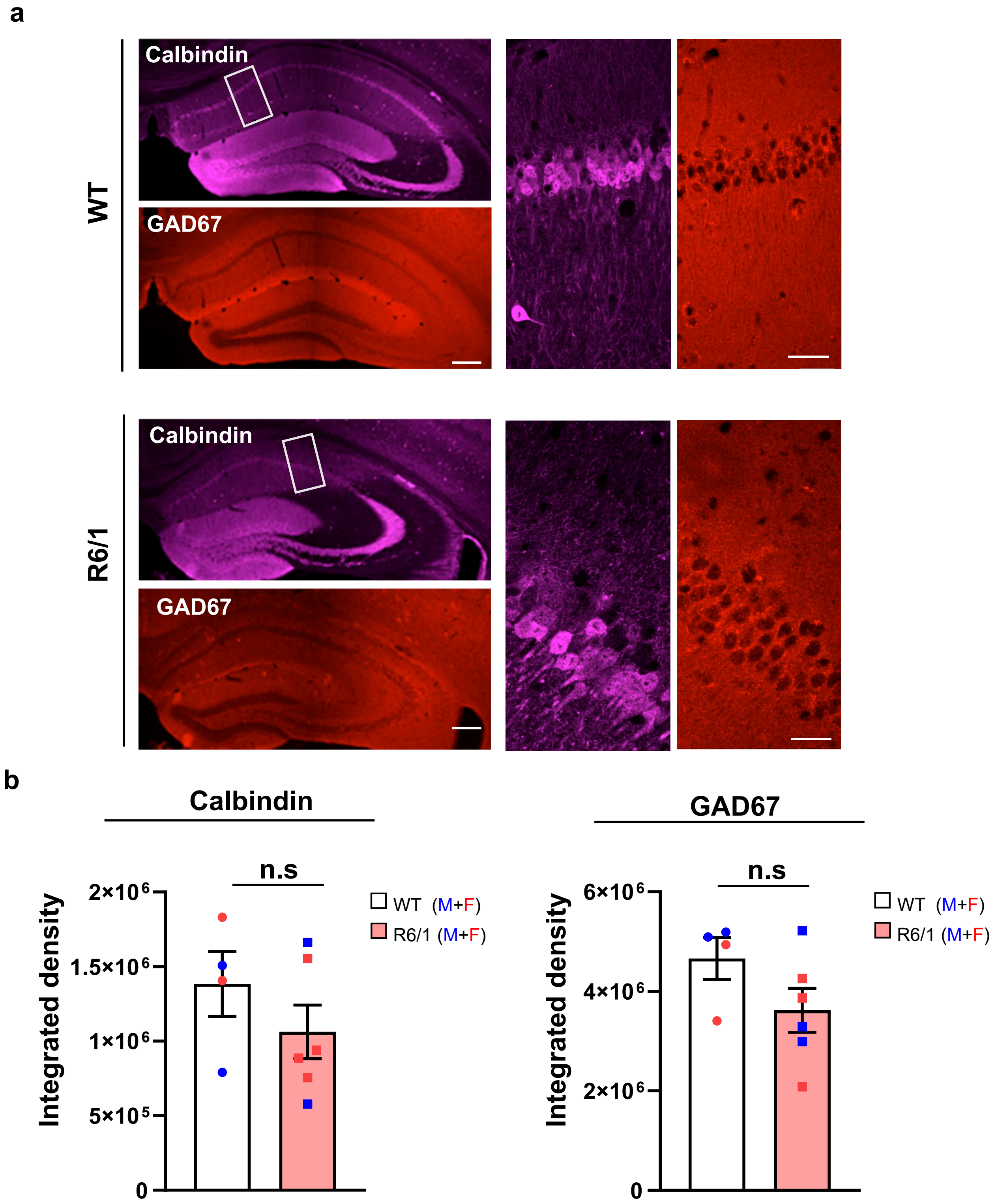

Figure S10. Characterization of calbindin and GAD67 expression in the hippocampus of WT and R6/1 mice. a Representative confocal images at low and high magnification showing the distribution of calbindin (purple) and GAD67 (red) in the hippocampus of naive WT and R6/1 mice. Scale bar = 200 µm (low magnification) and 25 µm (high magnification). Histograms display the integrated density of calbindin and GAD67 immunoreactivity. Data are presented as the mean ± standard error of the mean (SEM) with n= 4-6 mice per group. Statistical significance was assessed using the Mann-Whitney test.

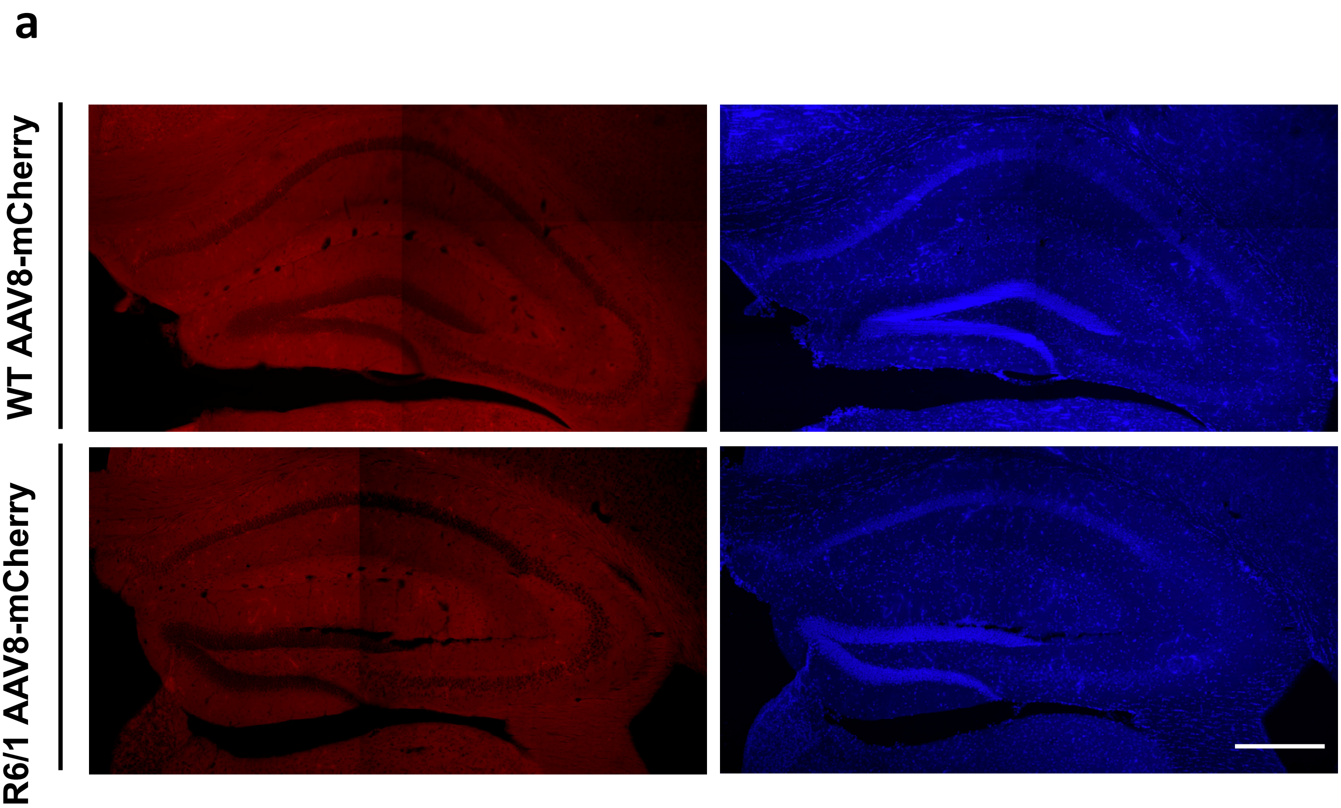
a
WT AAV8-mCherry
R6/1 AAV8-mCherry

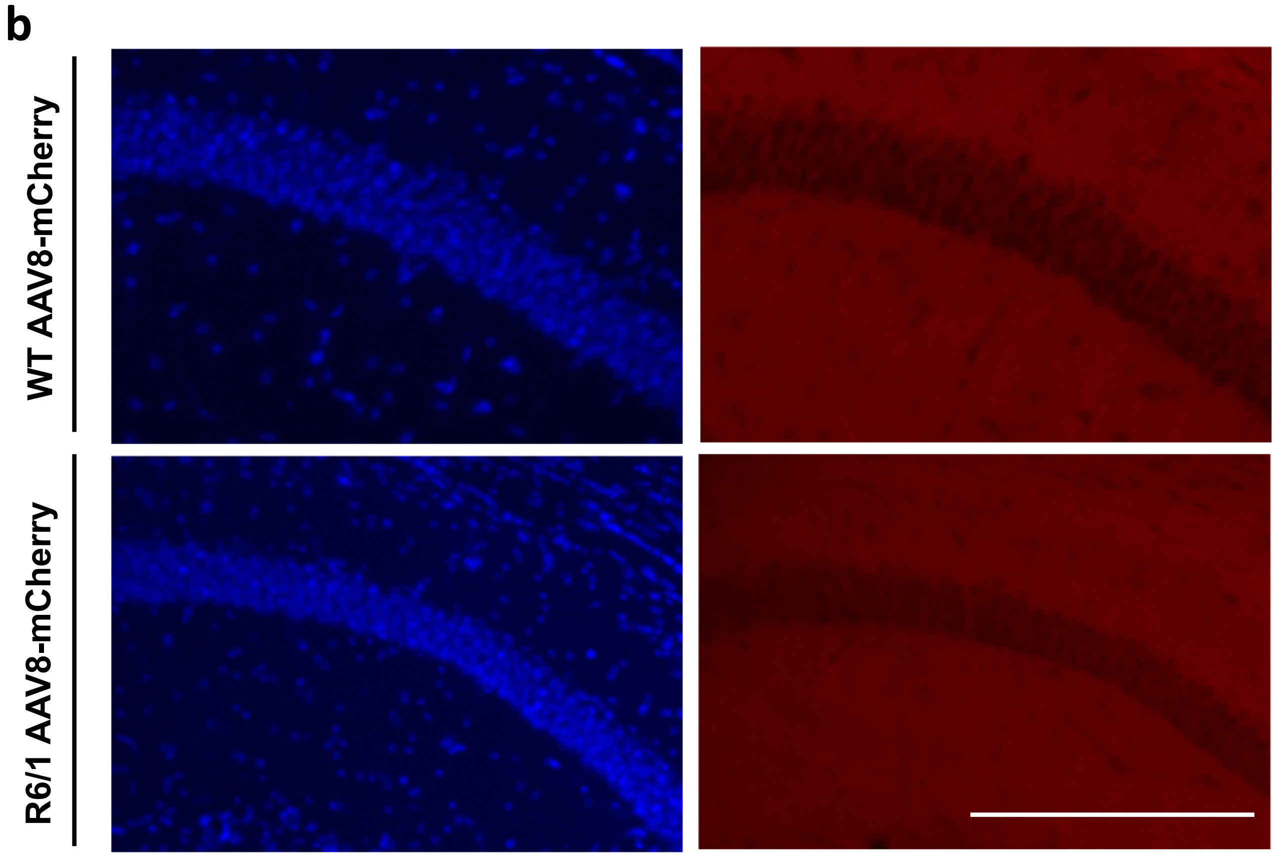
b
WT AAV8-mCherry
R6/1 AAV8-mCherry

Figure S11. Characterization of AAV8-mCherry transduction in the hippocampus of WT and R6/1 mice.

Representative confocal images showing AAV8-mediated mCherry transduction in the hippocampus of 13-week-old WT and R6/1 mice. Panel (**a**) displays low magnification images while panel (**b**) shows high magnification images highlighting the diffuse distribution of mCherry fluorescence in both genotypes. The images illustrate comparable transduction efficiency between WT and R6/1 mice. Scale bar are provided for each magnification (Scale bar=250μm).
