# Supplementary material for: Restoration of CB1 receptor function in hippocampal GABAergic neurons rescues memory deficits in Huntington’s disease models
Source: Transl Neurodegener. 2025 Aug 25;14:44. doi: 10.1186/s40035-025-00500-w (PMC12376758; doi:10.1186/s40035-025-00500-w)

Figura 1a

CB1R R6/1 males **Actin R6/1 males**

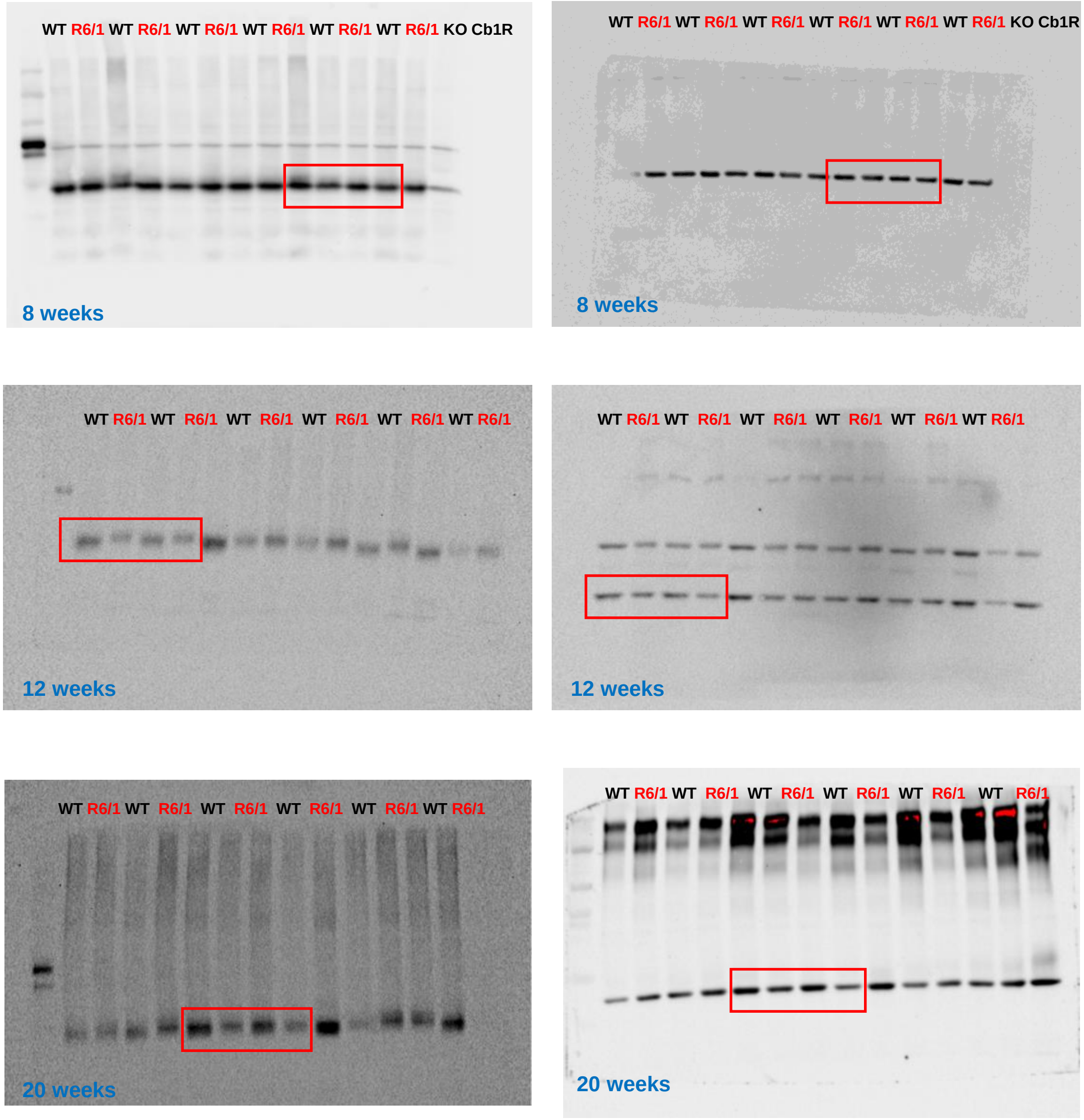

Figura 1a

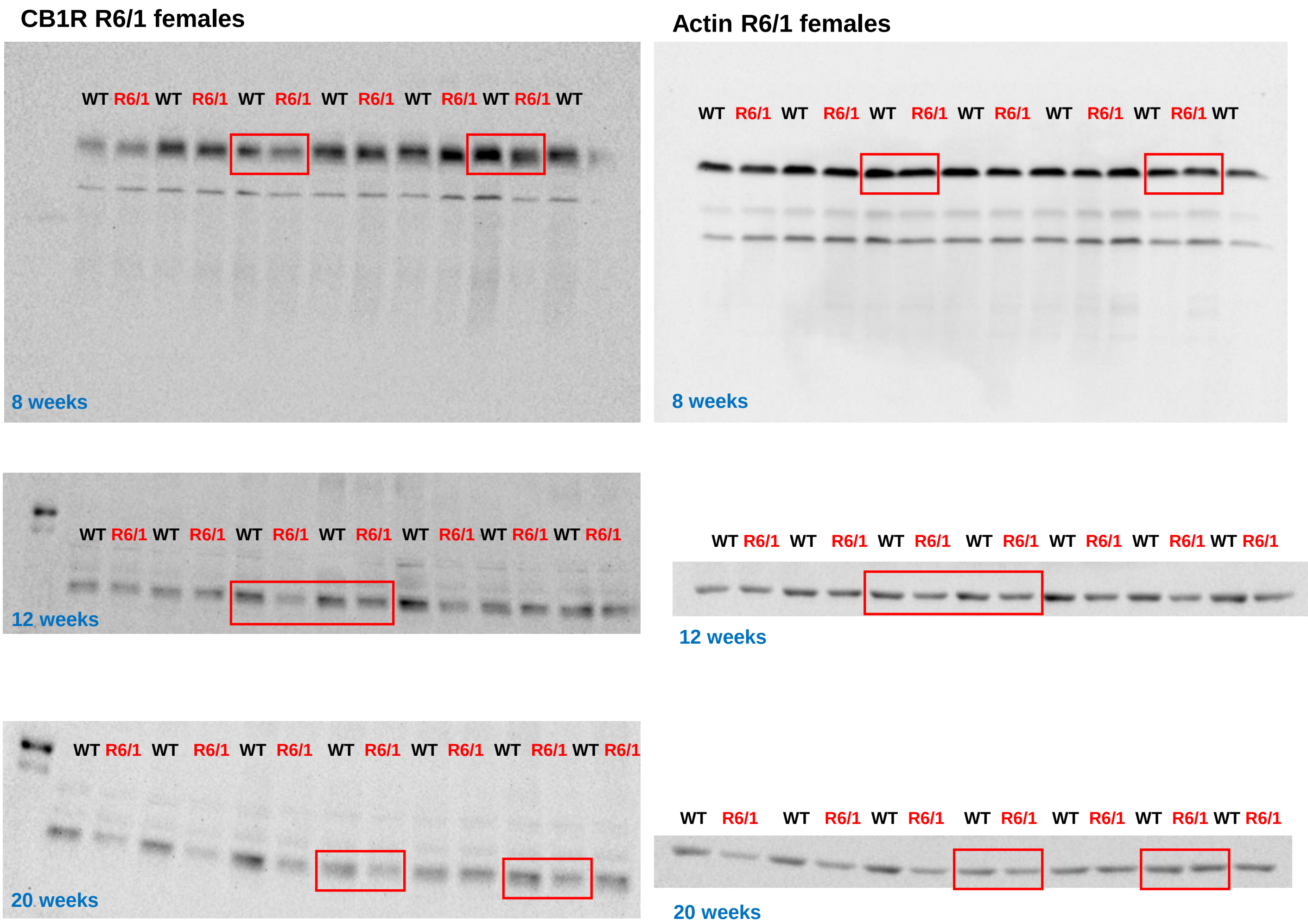
CB1R R6/1 females
WT R6/1 WT R6/1 WT R6/1 WT R6/1 WT R6/1 WT R6/1 WT
8 weeks
WT R6/1 WT R6/1 WT R6/1 WT R6/1 WT R6/1 WT R6/1 WT R6/1
12 weeks
WT R6/1 WT R6/1 WT R6/1 WT R6/1 WT R6/1 WT R6/1 WT R6/1
20 weeks
Actin R6/1 females
WT R6/1 WT R6/1 WT R6/1 WT R6/1 WT R6/1 WT R6/1 WT
8 weeks
WT R6/1 WT R6/1 WT R6/1 WT R6/1 WT R6/1 WT R6/1 WT R6/1
12 weeks
WT R6/1 WT R6/1 WT R6/1 WT R6/1 WT R6/1 WT R6/1 WT R6/1
20 weeks

Figura 3c

CB1R WIN R6/1 mice

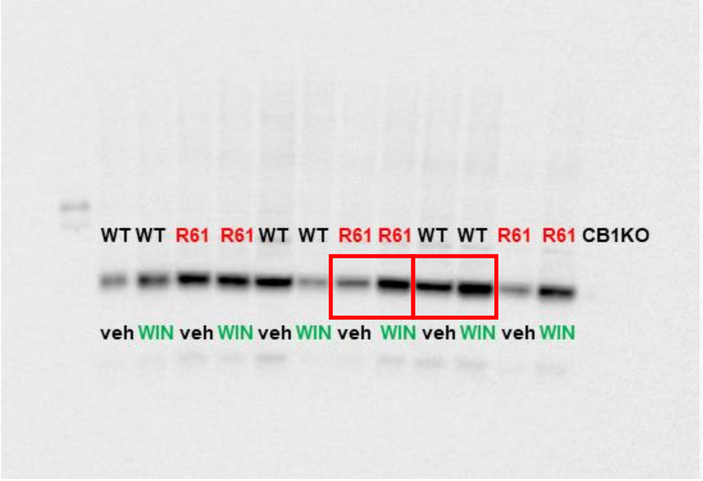

Actin

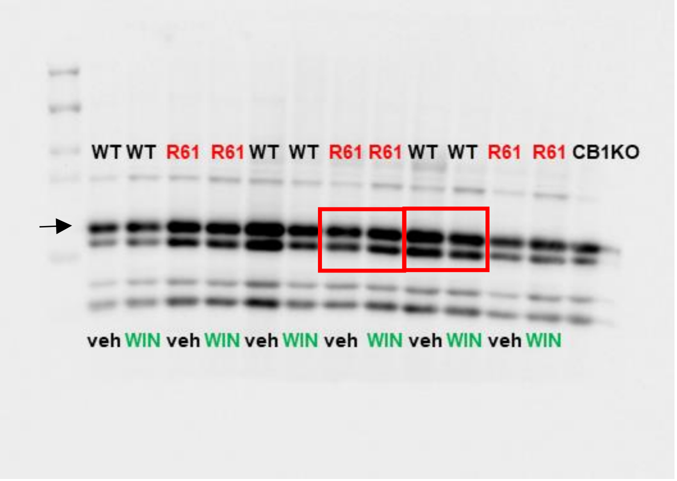

SUPPLEMENTARY FIGURES

Supplementary Figure 2

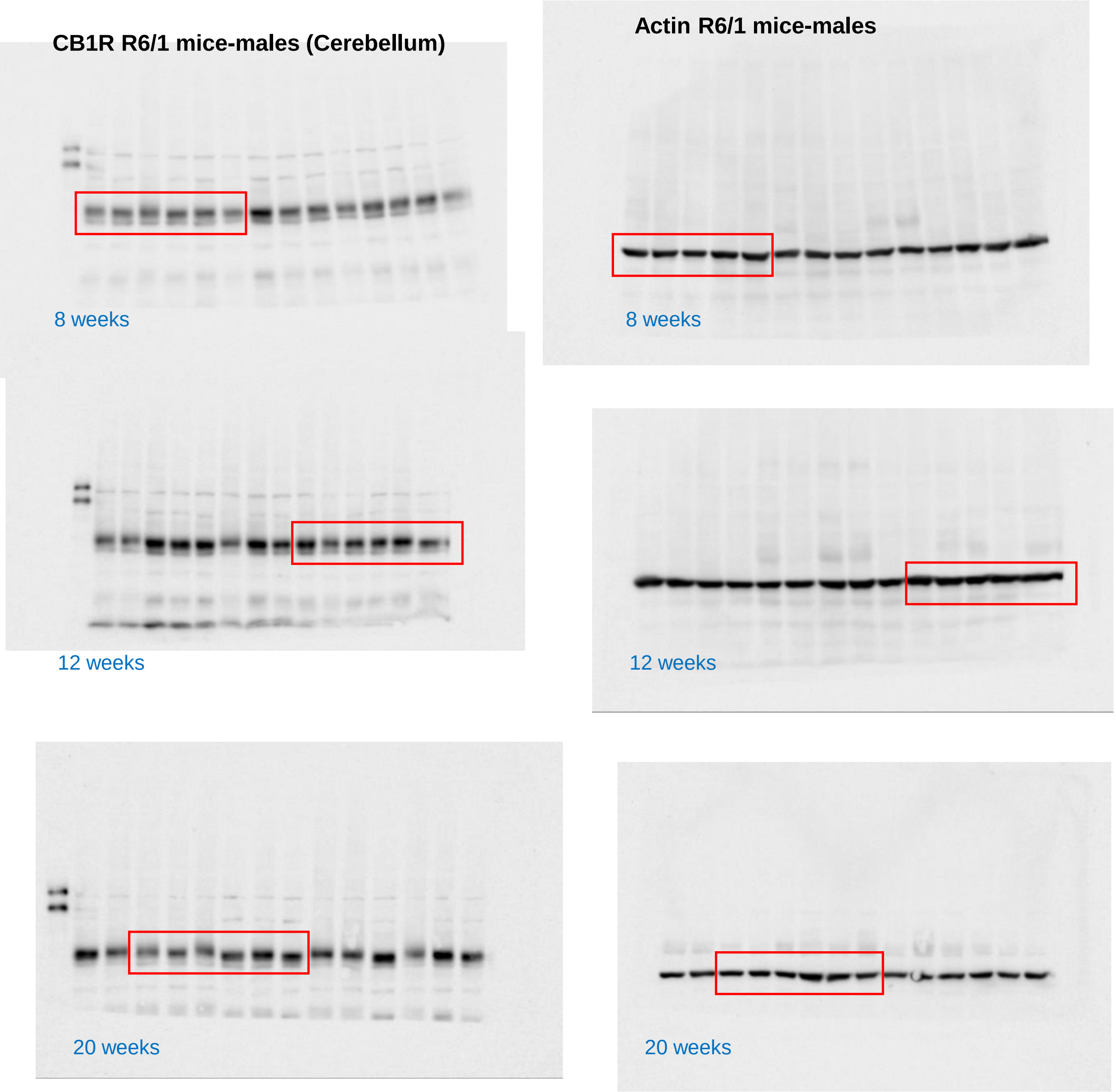

Supplementary Figure 2

CB1R R6/1 mice-females (Cerebellum)

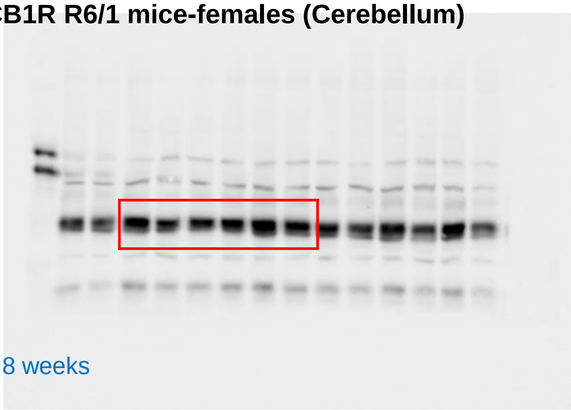

Actin R6/1 mice-females

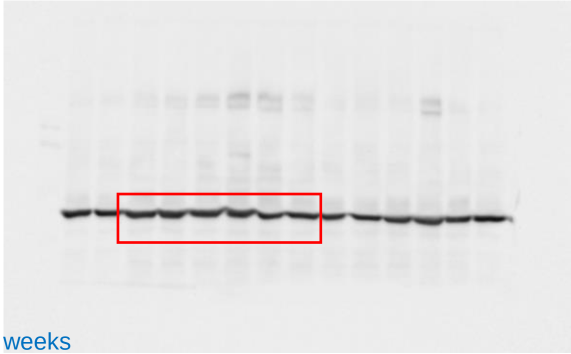

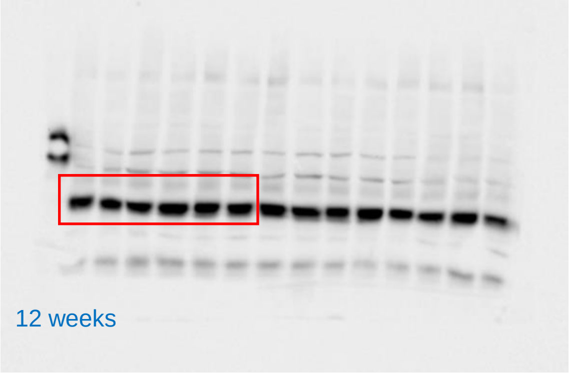

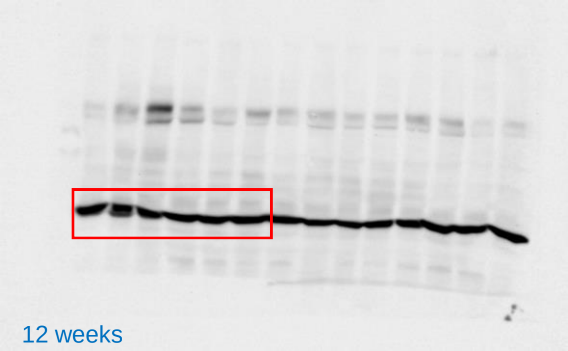

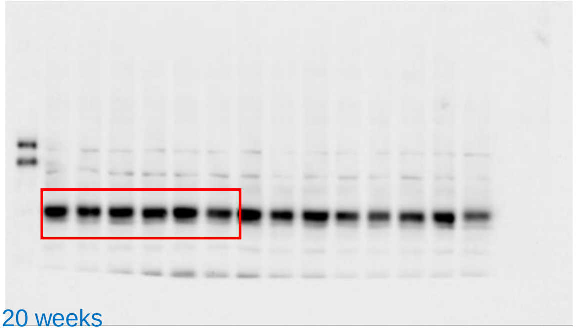

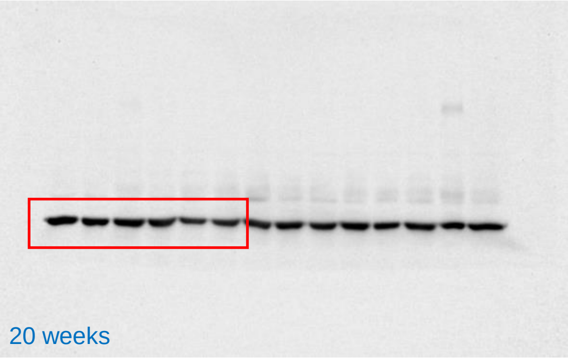

Supplementary Figure 4

Suppl Fig 5e

CB1R KI mice

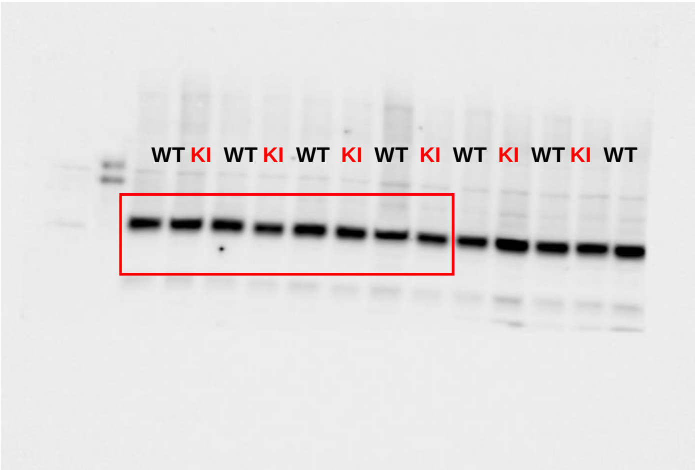

Actin

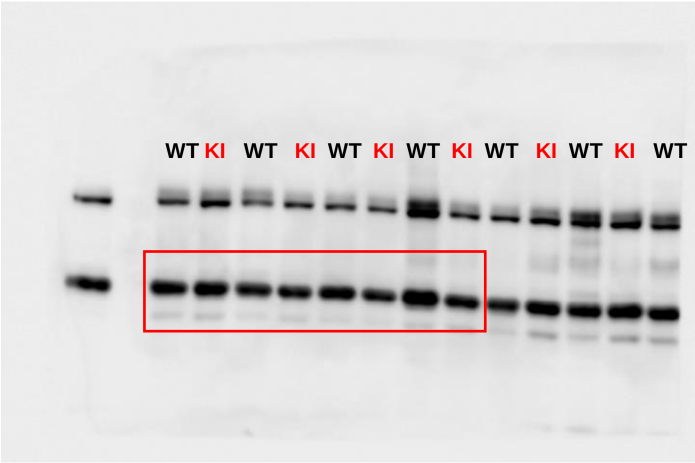

Suppl Fig 5f

CB1R KI mice WIN

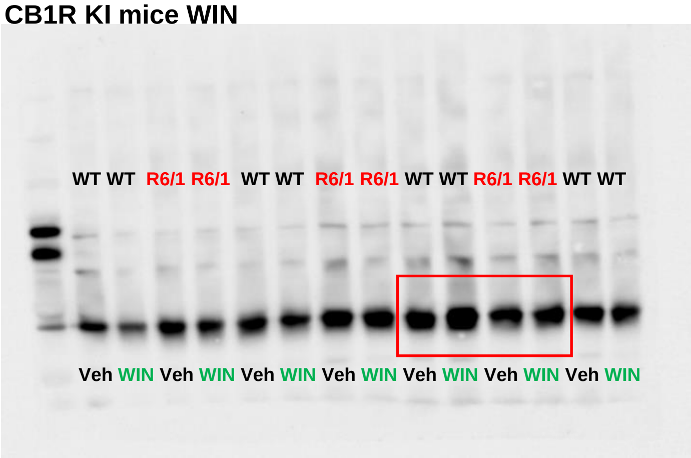

Actin

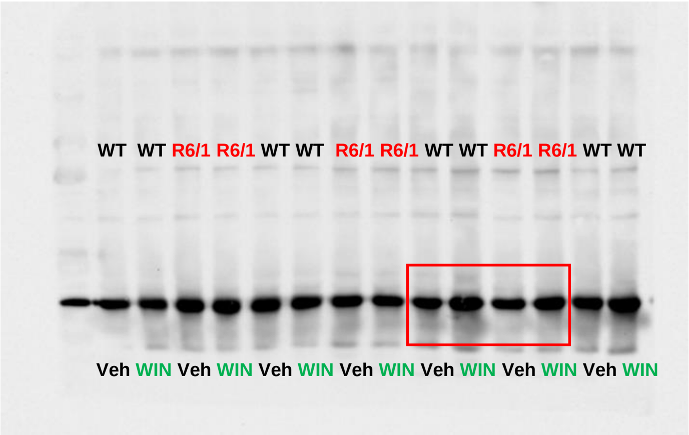

Supplementary Figure 7

Suppl Fig 7a

CB2R R6/1 males

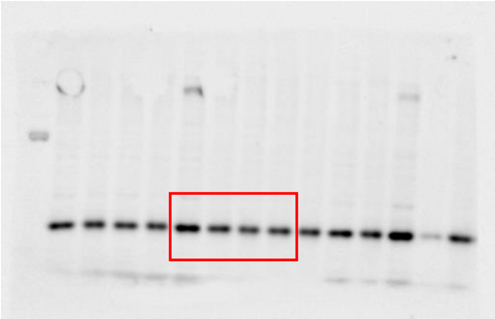

Actin

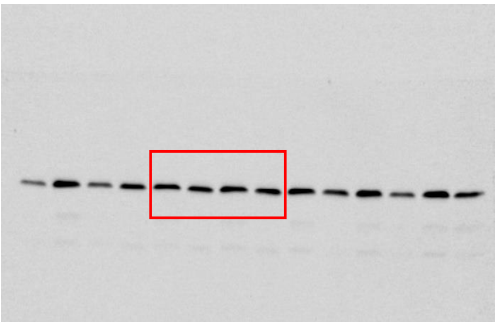

CB2R R6/1 females

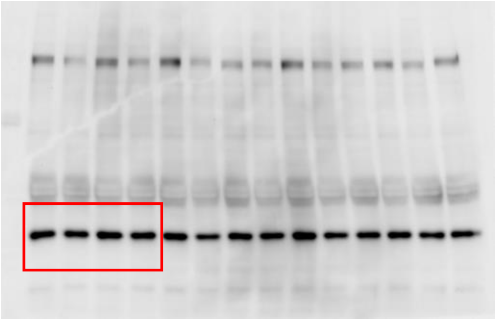

Actin

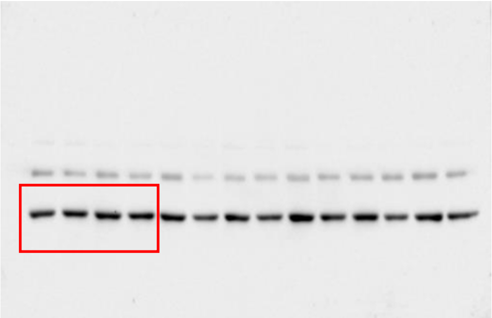

Suppl Fig 7b

CB2R KI males

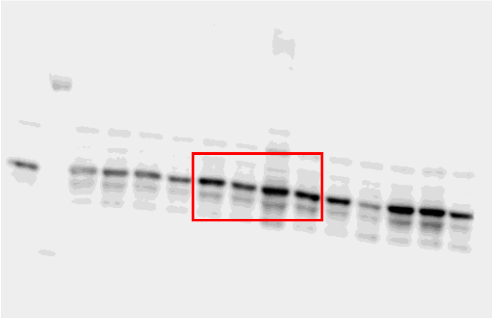

Actin

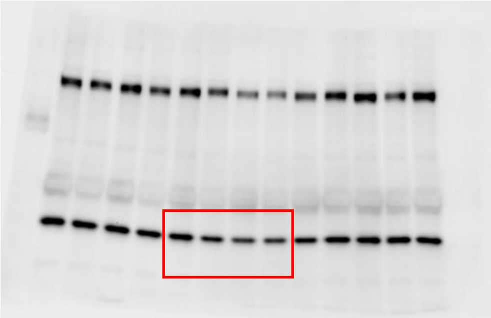

CB2R KI females

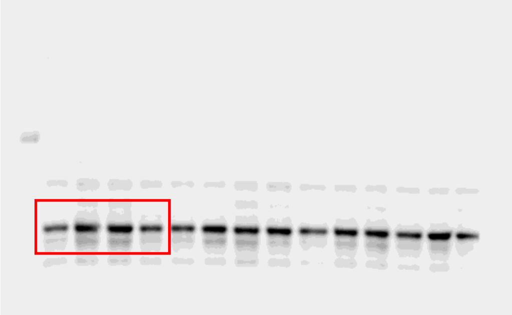

Actin

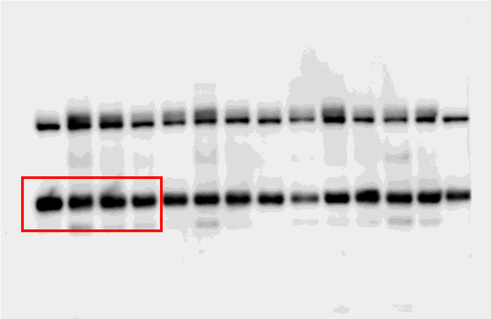

Supplementary Figure 7

Suppl Fig 7c

CB2R WIN R6/1 mice

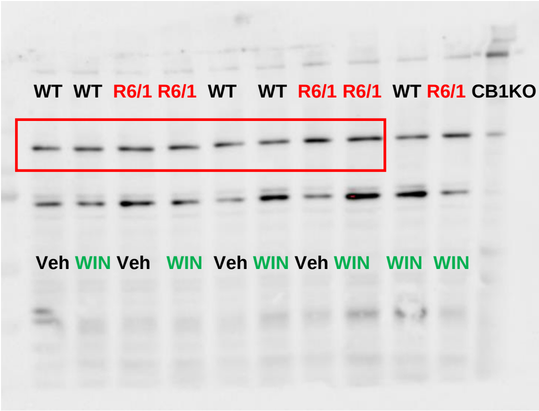

Actin

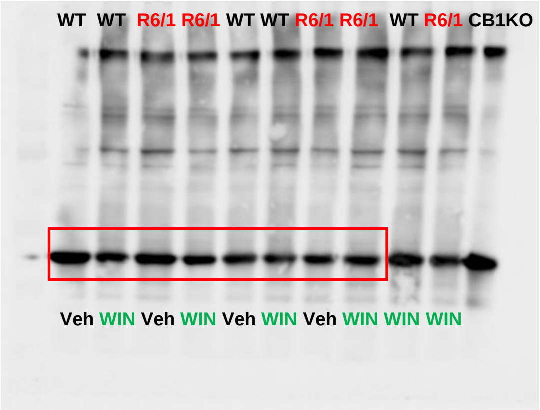

Suppl Fig 7d

CB2R WIN KI mice

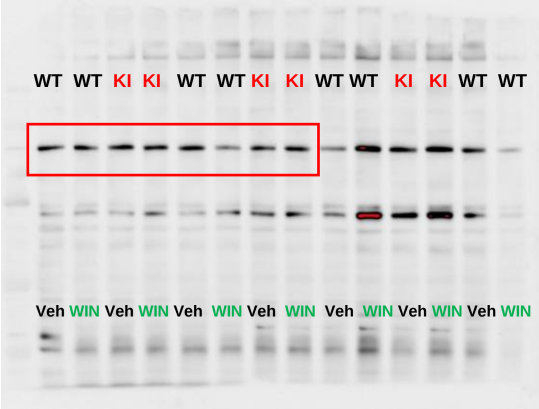

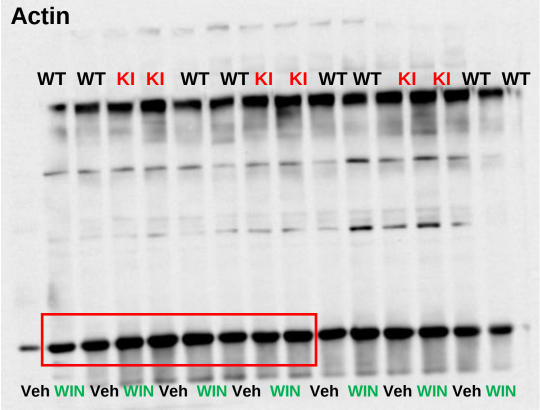

Supplementary Figure 8

PSD95

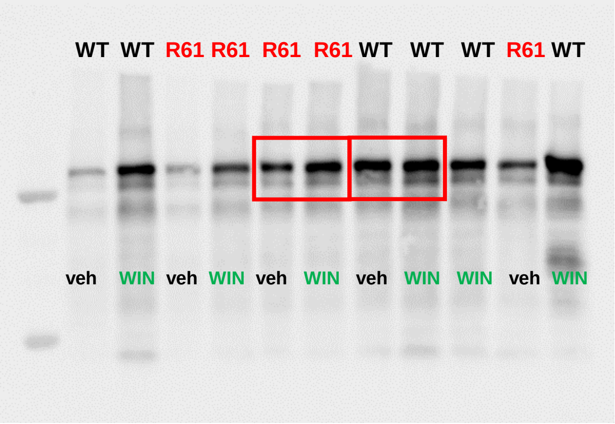

Actin

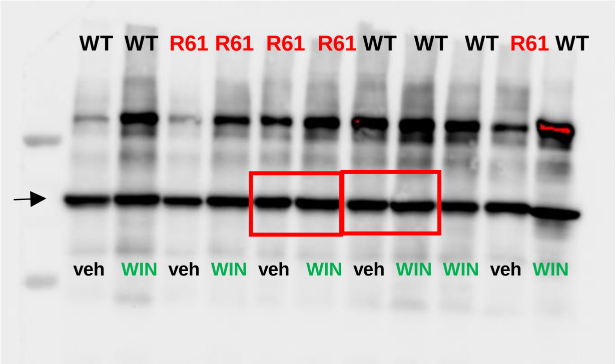

BDNF

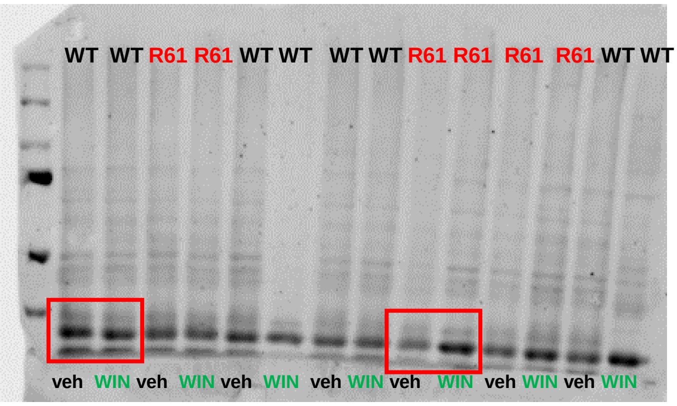

Actin

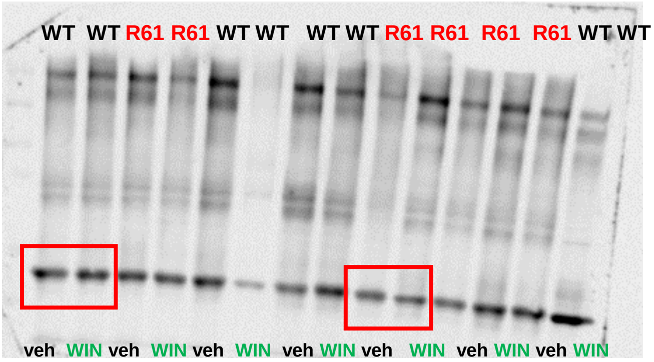

Supplement: Supplementary file 2 — Additional file 2. Uncropped Western blots. [file 40035_2025_500_MOESM2_ESM.pdf]
